# *Plectranthus ecklonii* Benth: A Comprehensive Review Into its Phytochemistry and Exerted Biological Activities

**DOI:** 10.3389/fphar.2021.768268

**Published:** 2021-11-30

**Authors:** Ana Ribeirinha Antão, Gabrielle Bangay, Eva María Domínguez-Martín, Ana María Díaz-Lanza, Patrícia Ríjo

**Affiliations:** ^1^ CBIOS -Research Center for Biosciences and Health Technologies, Universidade Lusófona de Humanidades e Tecnologias, Lisbon, Portugal; ^2^ University of Alcalá de Henares, Faculty of Pharmacy, Department of Biomedical Sciences, Pharmacology Area (Pharmacognosy Laboratory), New Antitumor Compounds: Toxic Action on Leukemia Cells Research Group, Campus University, Alcalá de Henares, Spain; ^3^ Instituto de Investigação do Medicamento (iMed.ULisboa), Faculdade de Farmácia, University of Lisbon, Lisbon, Portugal

**Keywords:** Plectranthus ecklonii, phytochemistry, pharmacology, bioactivity, plectranthus

## Abstract

**Ethnopharmacological Relevance:** *Plectranthus* genus (*Lamiaceae* family) contain several species with acknowledged ethnopharmacological uses, such as, for gastrointestinal and respiratory-related problems, due to their anti-inflammatory, antibacterial and antifungal properties. The bioactivity of isolated medicinal compounds from this genus justifies the increased interest in recent times for species of *Plectranthus*, placing them in the spotlight for natural product drug development.

**Aim of the study:** To the best of our knowledge, this is the first review on the biological activities of *Plectranthus ecklonii* Benth. As such, the aim of this review was three-fold: 1) to summarize the chemical compounds isolated from *P. ecklonii*; 2) to collate the biological activities and mechanisms of action of these compounds from *in vitro* studies; and 3) to evaluate the documented uses and potential applications of this species, in order to postulate on the direction of pharmaceutical uses of this species.

**Materials and methods:** An extensive database retrieval was performed using the electronic databases Web of Science, PubMed, Google Scholar and ScienceDirect. The search criteria consisted of the keywords “*Plectranthus ecklonii*”, “*Plectranthus ecklonii* + review”, “*Plectranthus ecklonii* + diterpenes” or “*Plectranthus ecklonii* + abietanes”, “*ecklonii* + parviflorone D”, searched individually and as combinations. Eligibility criteria were set out and titles in English, Portuguese and Spanish were reviewed, with all references included dating from 1970 to 2021. A total of 169 papers were selected and included. Chemical structures were drawn using ChemDraw 20.0, CID numbers were searched in PubChem and the PRISMA diagram was created using PowerPoint 2012.

**Results:** To date, a total of 28 compounds have been isolated from *P. ecklonii*, including diterpenes, triterpenes, flavonoids, and hydroxycinnamic acids. Most focused on the antimicrobial action of its constituents, although compounds have demonstrated other bioactivities, namely antioxidant, anti-inflammatory and antitumor. The most recent studies emphasize the diterpenoids, particularly parviflorone D, with the help of nanotechnology.

**Conclusions:** The widespread ethnobotanical and traditional uses of *P. ecklonii* can be scientifically justified by a range of biological activities, demonstrated by isolated secondary metabolites. These bioactivities showcase the potential of this species in the development of economically important active pharmaceutical ingredients, particularly in anticancer therapy.

## Introduction

Since ancient times, plants have been used for the prevention and treatment of a variety of ailments. Across different cultures, they have been the basis of traditional medicine practices and they continue to be important sources of drugs, especially in developing countries that still use herbal medicine as a first line of healthcare (Salim et al., 2008). Members of the *Lamiaceae* family are considered relevant, due to their therapeutic and culinary uses throughout the world (Srancikova et al., 2013).

*Plectranthus* spp. (Lamiaceae) have long been used in traditional medicine, likely due to the many bioactive compounds found in the genus, having several activities, such as anti-inflammatory, antimicrobial and antifungal (Abdel-Mogib et al., 2002; Lukhoba et al., 2006; de Albuquerque et al., 2007). These properties suggest *Plectranthus* as a likely genus of bioactive compounds suitable for medicinal drug development. The isolation and understanding of the secondary metabolites from *Plectranthus* species’ responsible for biological activity are important, not only to validate the popular common uses of this genus, but also to discover novel drug sources with important economic potential, or compounds that can be transformed into active ingredients.

The genus *Plectranthus* belongs to the Angiosperms family, Lamiaceae (Nepetoideae subfamily, Ocimeae tribe, Plectranthinae subtribe) and includes about 350 species, distributed mainly in subtropical Africa, Asia, and Australia (Dellar et al., 1996; Narukawa et al., 2001; Gaspar-Marques et al., 2008). The genus was first described by the French botanist L'Heritier in 1788 (Lukhoba et al., 2006) and, since then, the total number of species belonging to this genus has been increasing. Nowadays, *Plectranthus* spp. are known all over the world for their horticultural uses as they grow fast, produce beautiful flowers, and are resistant to most plant pests and diseases. *Plectranthus* spp. exist as herbs, sub bushes, or shrubs. In Europe, several species of *Plectranthus* are grown as ornamental plants (Abdel-Mogib et al., 2002). The potential medicinal and economic uses of *Plectranthus* spp. are of great interest. Hidden in this genus are potential treatments for many conditions. The most frequently cited use of *Plectranthus* spp. is for its medicinal properties. They have been used for different digestive disorders, skin and respiratory conditions, genitourinary infections, general infections and fever, pain, and musculoskeletal conditions (Narukawa et al., 2001; Abdel-Mogib et al., 2002; Lukhoba et al., 2006). Other applications include insect repellents, spells, and culinary herbs (Lukhoba et al., 2006; Pal et al., 2011). The main phytochemical constituents of the *Plectranthus* genus are diterpenes, phenolic compounds, and essential oils, the latter giving this genus its natural aroma (Abdel-Mogib et al., 2002; Rice et al., 2011).

**FIGURE 1:**
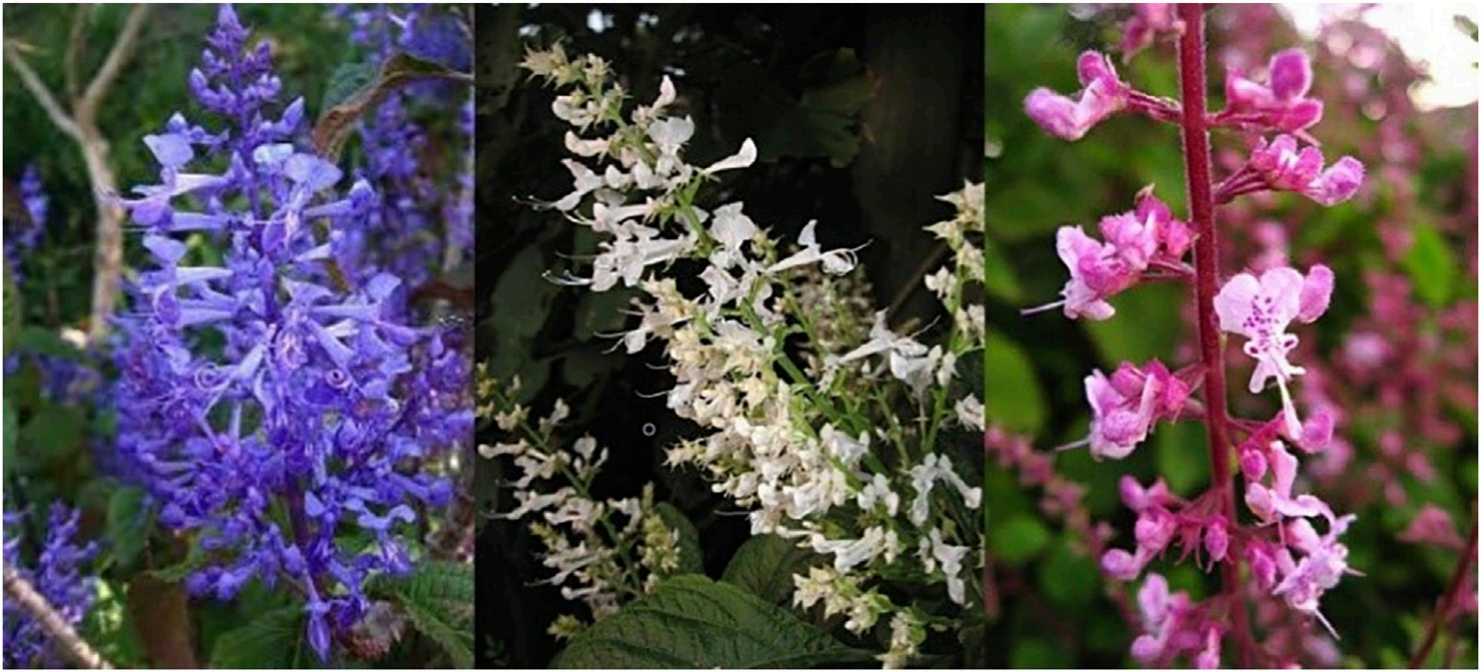
*Plectranthus ecklonii* “Medley-Wood” (blue), *P. ecklonii* “Tommy” (white), and *P. ecklonii* “Erma” (pink) (Van Jaarsveld, 2006).

The species *Plectranthus ecklonii* Benth. was first collected in 1813 by the naturalist William Burchel in the Eastern Cape. It is a fast-growing shrub, perennial or annual (1–3 m high), with ovate to elliptical leaves, arranged in pairs, and flowers from March to May, with a peak in April (Van Jaarsveld, 2006). It is easily propagated using cuttings or seeds and the young plants should be pruned after flowering, or at least before spring. There are three cultivable species available: the blue-flowered “Medley Wood”, the white-flowered “Tommy”, and the pink-flowered “Erma” (Figure 1). *P. ecklonii* is commonly known as Ecklon spur flower or Ecklon spoorsalie and is widely distributed in South Africa, Australia, New Zealand, Mexico, and the United States (Van Jaarsveld, 2006; Nyila et al., 2009).

*P. ecklonii* Benth. is traditionally used in South Africa to treat stomach aches, nausea, vomiting, and meningitis, symptoms usually associated with listeriosis infection (Lukhoba et al., 2006; Chassagne and Morgan, 2020). The leaves are used for tuberculosis-related problems and, in Zimbabwe, aerial parts are applied for skin diseases and skin hyperpigmentation problems. The activity of *P. ecklonii* against *Escherichia coli* justifies the use of *Plectranthus* spp. in traditional medicine for the treatment of gastrointestinal infections (Nyila et al., 2009). Similarly, the traditional use of this plant for skin infections may be related to the antibacterial activity of two of its diterpenes, parviflorone D (Salim et al., 2008) and parviflorone F (Srancikova et al., 2013), against *Staphylococcus aureus* (Simões et al., 2010).

**FIGURE 2:**
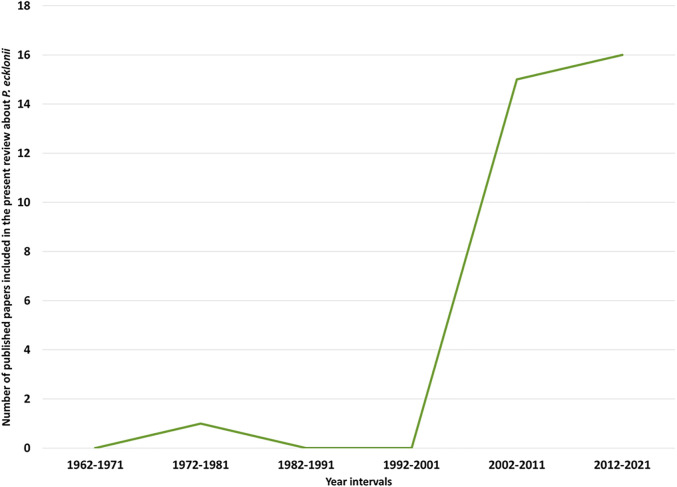
Number of published papers on *P. ecklonii* included in this review.

Since the 1960s, the number of papers published on *P. ecklonii* has been increasing, demonstrating the interest and importance of investigating this species. In fact, the number of published biological and chemical composition studies on *P. ecklonii* cited in this paper is 16 times higher in the years from 2012 to 2021 compared to that of the previous two decades (Figure 2). Growing intertest, lack of review paper on this species and recent developments in active antitumour compounds isolated from *P. ecklonii* justify and warrant a comprehensive up-to-date review. Consequently, the main aim of this review is to provide and evaluate the first complete compilation of the biological activities exerted by active compounds isolated, thus far, their mechanisms of action and, finally, offer an insight into their potential future use in natural product drug development.

## Methodology

For the preparation of this manuscript, an exhaustive bibliographic review among a variety of databases, including Google Scholar, PubMed, Web of Science and ScienceDirect was carried out to retrieve information on the phytochemical and pharmacological uses of *P. ecklonii*, up to January 2021. Books and other digital resources were also used, and key search terms included, “*Plectranthus ecklonii*”, “*Plectranthus ecklonii* + review”, “*Plectranthus ecklonii* + diterpenes” or “*Plectranthus ecklonii* + abietanes”, “*ecklonii* + parviflorone D”, among others. After collating all records relating to compounds isolated from the species *P. ecklonii*, the search was developed further on each compound individually, including studies on other *Plectranthus* species, and species belonging to the Lamiaceae family, were considered. Titles in English, Portuguese and Spanish were reviewed, and all references included dated from 1970 to 2021. A final total of 169 references were selected and included. Chemical structures were drawn using ChemDraw 20.0, CID numbers were searched in PubChem and the PRISMA flow chart was created using PowerPoint 2012.

## Isolated Compounds From *Plectranthus ecklonii* Benth

Plants produce a vast range of compounds originating from different biosynthetic pathways, with ranging molecular weights, which can be classified into different categories, such as primary and secondary metabolites. The relevance and application of secondary metabolites extends further than just medicine, including areas of agriculture and industry. Exploration into the different biosynthetic pathways and biological activities of these metabolites has led to the accepted categorization of their main, yet broad, categories of plant compounds: 1) terpenes or terpenoids, 2) alkaloids, and 3) phenolic compounds (Devappa et al., 2011). Terpenes are undoubtedly the largest, most distributed, and, from a structural point of view, the most diverse class of secondary metabolites. Their importance, particularly at the therapeutic level, justifies the numerous efforts made over the last few decades to clarify their biosynthesis (Devappa et al., 2011).

**FIGURE 3:**
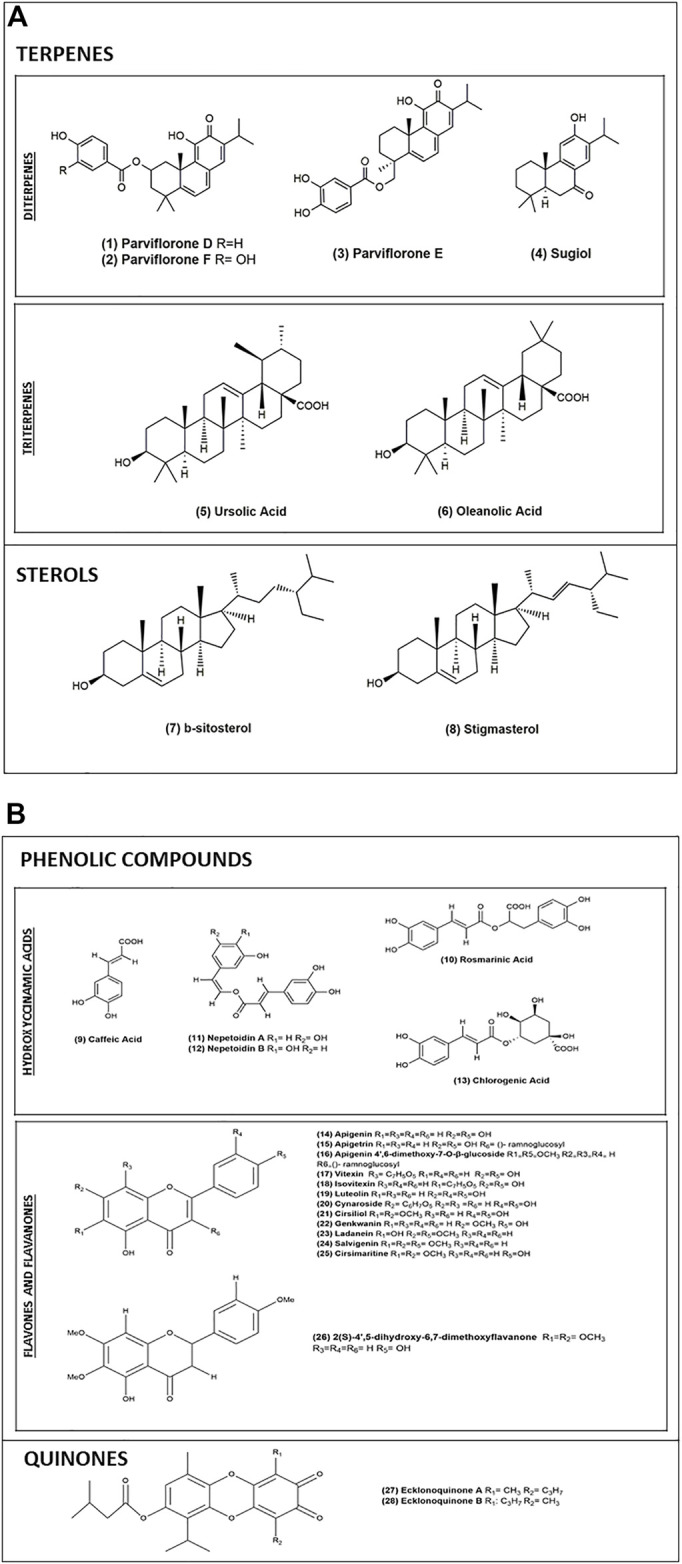
**(A)** Chemical structures of terpenes and sterols isolated from *P. ecklonii*. **(B)** Chemical structures of phenolic compounds and quinones isolated from *P. ecklonii*.

The main phytochemical constituents of the genus *Plectranthus* are diterpenes, essential oils, and phenolic compounds (Abdel-Mogib et al., 2002). Abietane diterpenoids of the species *Plectranthus* are specific antimicrobial and cytotoxic compounds (Teixeira et al., 1997; Gaspar-Marques et al., 2006). The main compound of the polar extract of *P. ecklonii* is rosmarinic acid (RA) (Rice et al., 2011), a common phenolic ester in the *Lamiaceae* family (Amoah et al., 2016). RA (Rice et al., 2011) together with two other esters of caffeic acid (CA) (Pal et al., 2011), nepetoidine A (Van Jaarsveld, 2006) and nepetoidine B (Nyila et al., 2009), are used as chemotaxonomic markers of the Nepetoideae subfamily (Grayer et al., 2003; Kubínová et al., 2013). Until now, a total of 28 compounds have been isolated from *P. ecklonii*, constituting a variety of different classes of plant compounds. In 1980, Uchida and colleagues were the first to report the isolation and identification of compounds from *P. ecklonii* (Uchida et al., 1980). At that time, they detected the presence of the abietane parviflorone F (Srancikova et al., 2013) and ecklonoquinones A (Śliwiński et al., 2020) and B (Andrade et al., 2018). After 40 years of research, the composition of this species is still not completely clear, however, here we have enumerated the compounds discovered thus far (Table 1 and Figure 3).

**TABLE 1:** The compounds isolated to date from *P. ecklonii* Benth.

	Isolated compounds	IUPAC name and CID number	Isolation and/or identification methods	Biological Activity^a^	Solvent extractor/plant part(s)	Ref.
	Terpenes and sterols					
Diterpenes	Parviflorone D **(1)**	11-hydroxy-2α-(4-hydroxybenzoyloxy)-abieta-5,7,9(11),13-tetraene-12-one **(1) [**101967011]	MS + NMR	Antibacterial	DCM:EtAc	Gurlal (2005)
			NMR	Antiplasmodic	DCM/ap	Van Zyl et al. (2008)
					EtAc/ap	Nyila et al. (2009), Nyila et al. (2012)
			MS + NMR	Antibacterial	Acetone/wp	Simões et al. (2010)
				Antitumour	Acetone	Martens and Mithöfer (2005), Ozgen et al. (2008), Falé et al. (2009), Bhatt et al. (2013), Kumar and Pandey, (2013), Petersen (2013), Burmistrova et al. (2015), Andrade et al. (2018), Costa et al. (2018), Sitarek et al., (2020), Śliwiński et al. (2020)
				Antioxidant		Wellsow et al. (2006)
			-	Enzyme inhibition	-	(Nyila et al., 2009; Dai and Mumper, 2010)
	Parviflorone F **(2)**	11-hydroxy-2α-(3,4-dihydroxybenzoyloxy)-abieta-5,7,9(11),13-tetraene-12-one **(2)** [10389067]	NMR	N/A	Ether/ap	Uchida et al. (1980)
				Antiplasmodic	DCM/ap	van Zyl et al. (2007), Van Zyl et al., (2008)
				Antibacterial	EtAc/ap	(Nyila et al., 2009; Nyila, 2010)
			NMR	Antitumour	EtAc/ap	Nyila et al. (2009)
			-	Antioxidant	-	Narukawa et al. (2001)
			-	Enzyme inhibition	-	Nyila et al. (2009), Dai and Mumper, (2010)
	Parviflorone E **(3)**	11-Hydroxy-19-(3,4-dihydroxybenzoyloxy)-abieta-5,7,9(11),13-tetraene-12-one **(3) [**10366501]	NMR	Antiplasmodic	ap	Van Zyl et al. (2008)
			HPLC-DAD/MS	Anticariogenic	Metanol/ap	Figueiredo et al. (2014)
			-	Antioxidant	-	Narukawa et al. (2001)
	Sugiol**(4)**	12-hydroxyabieta-8,11,13-trien-7-one **(4)** [94162**]**	MS + NMR	Antibacterial, antiplasmodic	Acetone/wp	van Zyl et al., (2007), Simões et al., (2010)
				Antioxidant		Chao et al. (2005), Bajpai et al. (2014)
				Antiinflammatory		Chao et al. (2005)
				Antitumoral		Son et al. (2005)
Triterpenes	Mix of Ursolic acid **(5)** and Oleanolic acid **(6)**	3β-hydroxy-urs-12-en-28-oic acid **(5) [**64945**]**		Antiinflammatory **(5)** Antibacterial, antiviral, antiplasmodic **(5 and 6)**, Antitumoral		Andersson et al. (2003), Checker et al. (2012)
		3β-hydroxyolean-12-en-28-oic acid **(6) [**10494**]**				
Sterols	Mix of *ß*-sitosterol **(7)** and Stigmasterol **(8)**	3β-stigmast-5-en-3-ol **(7) [**222284**]**		Antibacterial		Liu et al. (2019)
		stigmasta-5,22(*E*)-dien-3*ß*-ol **(8) [**5280794**]**				
	Phenolic compounds					
Hydroxyccinamic acids	Caffeic acid **(9)**	3,4-dihydroxycinnamic acid **(10) [**689043**]**	NMR	Antimicrobial	Methanol:Water/ap	Hawas et al. (2008)
			HPLC-DAD	Antioxidant, enzyme inhibition	Water (decoction)	Gomes et al. (2012)
			-	Antiinflammatory	-	Da Cunha et al. (2004), Gamaro et al. (2011), Yang et al. (2013)
			-	Antitumoral	-	Hawas et al. (2008), Gomes et al. (2012)
	Caffeic acid derivatives					
	Rosmarinic acid **(10)**	3,4-Dihydroxycinnamic acid (R)-1-carboxy-2-(3,4-dihydroxyphenyl)ethyl ester **(10) [**5281792**]**	N/A	Antibacterial	Water (decoction)/ap	Figueiredo et al. (2010)
			HPLC-DAD	Antioxidant, enzyme inhibition	Water (decoction)	Falé et al. (2009), Gomes et al. (2012)
			-	Antiinflammatory		Gamaro et al. (2011)
			-	Antitumoral		Figueiredo et al. (2010)
	Nepetoidin A **(11)**	(Z,E)-[2-(3,5-dihydroxyphenyl)ethenyl] 3-(3,4-dihydroxyphenyl)-2-propenoate **(11) [**5316820**]**	HPLC-DAD/NMR	Anticariogenic	Methanol/ap	Figueiredo et al. (2014)
				Antifungal and antioxidant	Diethyl ether/ap	Grayer et al. (2003)
	Nepetoidin B **(12)**	(Z,E)-[2-(3,4-dihydroxyphenyl)ethenyl] 3-(3,4-dihydroxyphenyl)-2-propenoate **(12)** [5316819]		Anti-inflammatory (**11** and **12**)	-	Grayer et al. (2003)
	Chlorogenic acid **(13)**	5-*O*-cafeoilquinic acid **(13) [**1794427**]**	HPLC-DAD	Antioxidant, enzyme inhibition	Water (decoction)	Gomes et al. (2012)
Flavones	Apigenin **(19)**	4’,5,7-trihydroxyflavone **(19) [**5280443**]**	NMR	Antimicrobial	Methanol:Water/ap	Hawas et al. (2008)
			HPLC/LC-MS	N/A	Diethyl ether/ap	Grayer et al. (2010)
			No information provided	Antiplasmodic	-	Lehane and Saliba, (2008)
			-	Antioxidant	-	Velioglu et al. (1998), Pietta (2000)
			-	Anti-inflammatory	-	Nakanishi et al. (1990), Hirano et al., (2004), Kim et al. (2004), Choi et al. (2014), Lago et al. (2014)
			-	Antitumoral	-	Wu et al. (2014)
	Apigenin derivatives					
	Apigetrin **(15)**	apigenin 7-O-*β*-glucoside **(15) [**5280704**]**	NMR	Antimicrobial	Methanol:Water/ap	Hawas et al. (2008)
	Apigenin 4',6-dimethoxy -7-O-β-glucoside **(16)**	apigenin 4',6-dimethoxy-7-O-*β*-glucoside **(16) [**44257792]				
	Vitexin **(17)**	Apigenin-8-*C-*glucoside **(17) [**5280441**]**	-	Antioxidant	-	Borghi et al. (2013)
	Isovitexin **(18)**	Apigenin-6-*C-*glucoside **(18) [**162350**]**	NMR	Antimicrobial	Methanol:Water/ap	Lin et al. (2002), Choi et al. (2014)
	Luteolin **(19)**	3′,4′,5,7-tetrahydroxyflavone **(19) [**5280445**]**	-	Anti-inflammatory	-	Kim et al. (1999), Lin et al. (2008), Chen et al. (2014)
			-	Antitumoral	-	Lin et al. (2008)
	Cynaroside **(20)**	luteolin 7-*O*-glucoside **(20) [**5280637**]**	NMR	Antimicrobial, antioxidant	Methanol:Water/ap	Hawas et al. (2008)
						Odontuya et al. (2005)
	Cirsiol **(21)**	6-Hydroxyluteolin 6,7-dimethyl ether **(21) [**160237**]**	-	Antitumoral (acts as radiosensitizer)	-	Kang et al. (2013)
	Genkwanin **(22)**	4',5-Dihydroxy-7-methoxyflavone **(22) [**5281617**]**	HPLC/LC-MS	N/A	Diethyl ether/ap	Grayer et al. (2010)
	Ladanein **(23)**	scutellarein-5,7,4’-trimethyl ether **(23) [**3084066**]**				
	Salvigenin **(24)**	Scutellarein 6,7,4’-trimethylether **(24) [**161271**]**		Antioxidant		Grayer et al. (2010)
	Cirsimaritin **(25)**	Scutellarein 6,7-dimethylether **(25) [**188323**]**		N/A		Grayer et al. (2010)
Flavanone	2(S)-4',5-dihydroxy-6,7-dimethoxyflavanone **(26)**	2(S)-4',5-dihydroxy-6,7-dimethoxyflavanone **(26) [**14078484**]**	NMR		Ether/ap	Uchida et al. (1980)
	Quinones					
	Ecklonoquinone A **(27)**	[4,6-dimethyl-7,8-dioxo-1,9-di(propan-2-yl)dibenzo-p-dioxin-2-yl] 3-methylbutanoate **(27) [**21576878**]**	NMR	N/A	Ether/ap	Uchida et al. (1980)
	Ecklonoquinone B **(28)**	[4,9-Dimethyl-7,8-dioxo-1,6-di(propan-2-yl)dibenzo-p-dioxin-2-yl] 3-methylbutanoate **(28) [**21576879**]**				

N/A, Not Applicable; ap, aerial parts (leaves); wp, the whole plant.

HPLC, High performance liquid chromatography; LC-MS, Liquid Chromatography-Mass Spectrometry; NMR, Nuclear Magnetic Resonance.

a Biological activity presented in table is related to the compound (can be isolated from P. ecklonii and other species)

Except for one study, in which the whole plant (wp) was used (Simões et al., 2010), all other studies reported using aerial parts (leaves), possibly to mimic more faithfully the traditional use of this plant. Besides, the harvesting of leaves for medicinal purposes is more sustainable than that of other parts of the plant, such as roots and stems, whose excessive harvesting could even threaten the survival of the plant (Zschocke et al., 2000).

### Diterpenes

Diterpenes, a heterogeneous class of natural compounds based on a skeleton with 20 carbon atoms (C_20_), are the most common group of secondary compounds in the genus *Plectranthus*, most of which are highly modified abietanoids containing phenolic or quinone rings, in addition to some labdanes, ent-kaurenes, and seco-kaurenes (Abdel-Mogib et al., 2002). Regarding the type of hydrocarbon skeleton, diterpenes can be acyclic or cyclic. Most belong to the cyclic group, and it is precisely the diversity in the cyclization of the hydrocarbon skeleton, combined with the diversity of functional groups with oxygen (e.g., hydroxyl, carbonyl, epoxides, quinones, acids, and acid derivatives) which defines their multiple biological properties (Wang et al., 2002; Rijo et al., 2013). In general, diterpenes are compounds with medium to low polarity, although, they often occur in plants in a glycosylated form, in which case, they are polar substances. Medium polar solvents such as dichloromethane (DCM), ethyl acetate (EtOAc), and acetone are usually used for their extraction, or strong polar solvents, such as methanol, mixtures of alcohols and water, or even pure water (Waksmundzka-Hajnos and Sherma, 2011).

From the ethyl acetate extract of *P. ecklonii*, two abietanes, parviflorone D (Salim et al., 2008) and parviflorone F (Srancikova et al., 2013) have been isolated (Nyila et al., 2009). In 2008, in a study published by Van Zyl and colleagues, these two abietanes were also isolated from a DCM extract of *P. ecklonii* (Van Zyl et al., 2008). The detection of parviflorone E (Abdel-Mogib et al., 2002) required a stronger polar solvent, in this case, methanol (Figueiredo et al., 2014). In another study, parviflorone D (Salim et al., 2008) was isolated from an acetonic extract of *P. ecklonii*, together with the diterpene sugiol (de Albuquerque et al., 2007) and mixtures of ursolic acid (UA) (Lukhoba et al., 2006) with oleanolic acid (OA) (Dellar et al., 1996) and ß-sitosterol (Gaspar-Marques et al., 2008) with stigmasterol (Narukawa et al., 2001) (Simões et al., 2010). Meanwhile, the compounds OA (Dellar et al., 1996), ß-sitosterol (Gaspar-Marques et al., 2008), and stigmasterol (Narukawa et al., 2001) have also been isolated in *Plectranthus bishopianus* Benth., but from a methanolic extract (Syamasundar et al., 2012).

Interest in diterpenoid isolation continues to grow due to its wide range of biological activities (Hanson, 2005). Abietane diterpenoids have attracted interest on account of their antibacterial (Dellar et al., 1996; Teixeira et al., 1997; Figueiredo et al., 2014), antioxidant (Rijo et al., 2009) and insect antifeedant activities (Wellsow et al., 2006), as well as their inhibitory effects on different human cancer cell lines (Marques et al., 2002). Abietane is the skeleton with the highest occurrence and most widespread in *Lamiaceae* (Vestri Alvarenga et al., 2001). Abietane diterpenoids account for the most common secondary metabolites in *Plectranthus*. Abietanoids in *Plectranthus* mostly consist of royleanones, spirocoleons, and quinines (Abdel-Mogib et al., 2002). In 2007, Van Zyl and colleagues isolated seven abietane diterpenes, including parviflorone D (Salim et al., 2008) and F (Srancikova et al., 2013), from the leaves of five different *Plectranthus* species (van Zyl et al., 2007).

### Triterpenes

Triterpenes and sterols are two groups genetically engineered from the same precursor, squalene. Triterpenes, with the molecular formula C_30_H_48_, belong to the terpene group and may have acyclic carbon skeletons or contain mono-, bi-, tri-, tetra-, and pentacyclic structures (Dewick, 2002; Xu et al., 2004). From a biological point of view, the most important triterpenoid structures are those with the carbon skeletons of dammarane and euphane (tetracyclic triterpenes), oleanane, ursane, and lupane (pentacyclic triterpenes) (Dzubak et al., 2006).

For a long period of time, triterpenes were disregarded due to their low hydrophilicity. However, multiple studies, supporting their broad range of pharmacological activities and beneficial effects against several types of human diseases, including cancers, has been emerging (Patlolla and Rao, 2012). The chemistry of oleanane- and ursane-type triterpenoids have been actively explored in recent years, and their biological and pharmacological activities have been found to span a variety of properties, namely, antitumor, anti-viral, anti-inflammatory, hepato- and gastroprotective, antimicrobial, antidiabetic, and haemolytic properties (Sun et al., 2006). These triterpenoids are relatively non-toxic but their structural similarity to cholesterol gives them low water solubility, a major disadvantage in terms of bioavailability and, therefore, reduced therapeutic potential (Soica et al., 2014). However, studies of structure activity relationships (SAR) have shown that modifications in certain areas of the nuclei of these compounds can lead to significantly more active new derivatives (Sun et al., 2006). In Asian countries, the traditional applications of plants containing OA (Dellar et al., 1996) or UA (Lukhoba et al., 2006) in folk medicine are also multiple, including for anti-inflammatory, analgesic, sedative, hepatoprotective and cardiotonic effects (Liu, 1995; Poolier and Goossens, 2012). Other studies have also demonstrated their antioxidant, antiallergic, antipruritic, and antimicrobial potential (Jesus et al., 2015). For example, plant-based medicines with UA (Lukhoba et al., 2006) and OA (Dellar et al., 1996) are widely used in the treatment and prevention of type II *diabetes mellitus* in Traditional Chinese Medicine (TCM) and Indian medicines (Wang et al., 2013).

In the *Plectranthus* genus, common triterpenes have been isolated, such as UA (Lukhoba et al., 2006) OA (Dellar et al., 1996), betulin, and betulinic acid. Triterpenic acids exhibit important biological and pharmacological activities, including anti-inflammatory, antimicrobial, antiviral, cytotoxic, and cardiovascular effects (Lin et al., 2002; Odjakova et al., 2012). UA (Lukhoba et al., 2006) and OA (Dellar et al., 1996) are isomeric triterpenic acids that only differ in the position of the methyl (CH_3_) group on C_29_ and always exist simultaneously in the same plant (Xu et al., 2004). In 1971, Misra and colleagues reported the isolation of triterpenes UA (Lukhoba et al., 2006), OA (Dellar et al., 1996) from the methanolic extract of *P. bishopianus* Benth., which are also found in *P. ecklonii* (Misra et al., 1971; Andrade et al., 2021).

One of the traditional uses of *P. ecklonii* is for skin ailments and, in recent years, collagenase inhibitors, compounds that prevent the enzymatic degradation of the dermal matrix, have been identified in extracts of *Plectranthus* spp*.* as OA (Dellar et al., 1996) and UA (Lukhoba et al., 2006). In organic extracts of *P. ecklonii,* high collagenase inhibition has been reported and, further to this, the isolated compounds, OA (Dellar et al., 1996), and UA (Lukhoba et al., 2006), demonstrated higher anti-elastase activity when compared to the extract. This is most probably due to the compounds binding to the catalytic site of the enzyme, justifying its use in dermatology and cosmetics (Andrade et al., 2021). As they share similar structural features, OA (Dellar et al., 1996) and its isomer, UA (Lukhoba et al., 2006), frequently occur simultaneously (Jesus et al., 2015).

### Phytosterols

Phytosterols or plant sterols are fatty acids contained in plants. Their nutritional interest stems from their structural similarity to cholesterol (Van Jaarsveld, 2006), and their ability to lower plasma cholesterol and low-density lipoprotein (LDL) levels. Unlike sterols, triterpenes do not occur in the animal kingdom (Gabay et al., 2010). In recent decades, phytosterols have received much attention due to their capability to inhibit intestinal cholesterol absorption, resulting in lower total serum cholesterol and LDL cholesterol levels (Feng et al., 2017). *β*-sitosterol (Gaspar-Marques et al., 2008) and stigmasterol (Narukawa et al., 2001) are the most abundant plant sterols and occur in complex mixtures. The nutritional interest in sterols is due to their similarity in structure to cholesterol (Gabay et al., 2010).

Various activities have been attributed to *β*-sitosterol (Gaspar-Marques et al., 2008), including anti-hyperlipidaemia, anti-inflammatory and anti-tumoral. Some studies suggested that β-sitosterol (Gaspar-Marques et al., 2008) could be used as an antibacterial agent and possess the ability to protect the gastric mucosa from acetic acid- or aspirin-induced damage (Liu et al., 2019). In 2017, Feng and colleagues reported less severity of mucosal colitis in mice treated with β-sitosterol (Gaspar-Marques et al., 2008) and stigmasterol (Narukawa et al., 2001) (Feng et al., 2017). *ß*-sitosterol (Gaspar-Marques et al., 2008) is used as a herbal treatment for benign prostatic hyperplasia. This application is described in the literature in four randomized, placebo-controlled, double-blind studies and included a total of 519 men. Three of the studies reported significant benefits in the perception of symptoms and measurable parameters, such as urinary flow rate. In one study, during an 18-months follow-up period, the beneficial effects of treatment with *β*-sitosterol were maintained (Berges et al., 2000). However, further clinical trials are needed to establish the real efficacy and long-term effects of *ß*-sitosterol (Gaspar-Marques et al., 2008). Stigmasterol (Narukawa et al., 2001) is used in several chemical processes, which are designed to yield numerous synthetic and semi-synthetic compounds for the pharmaceutical industry. It acts as a precursor in the synthesis of progesterone and as an intermediate in the biosynthesis of androgens, oestrogens, corticoids, and in the synthesis of vitamin D3 (Sandhar et al., 2011). Although most studies have focused on the cholesterol-lowering activity of stigmasterol (Narukawa et al., 2001), other bioactivities have been attributed to this plant sterol compound, one of which is a potential anti-inflammatory effect (Gabay et al., 2010). In a more recent study, *β*-Sitosterol (Gaspar-Marques et al., 2008) and stigmasterol (Narukawa et al., 2001) did not demonstrate anti-inflammatory responses through the NO scavenging pathways, however, further studies on the response through other mechanisms, such as COX-2, should be explored to identify the mediators responsible for the anti-inflammatory effect (Andrade et al., 2018). Parviflorone D (Salim et al., 2008) in mixtures of plant sterols, such as *β*-sitosterol (Gaspar-Marques et al., 2008) and stigmasta-5,22(*E*)-dien-3*ß*-ol (Narukawa et al., 2001), have been isolated from *P. ecklonii* (Simões et al., 2010)**.**

### Phenolics

Phenolic compounds are important plant secondary metabolites that play a key role in disease-resistance, pest protection, and species dissemination. They are widespread constituents of plant foods (fruits, vegetables, cereals, chocolate, etc.) and beverages (tea, coffee, beer, wine, etc.). There are ten main classes of phenolic compounds, which includes phenolic acids, flavonoids, and tannins, and are generally involved in the defence against ultraviolet (UV) radiation or aggression by pathogens, parasites, and predators, as well as contributing to plants’ colours (Dai and Mumper, 2010). Flavonoids and phenolic acids (mainly hydroxycinnamic acids) are the most abundant compounds found in plant extracts (Ramu et al., 2012). The biological effects of hydroxycinnamic acids in humans are mainly related to their antioxidant function, although many other bioactivities have been reported for these compounds, such as antidiabetic, antigenotoxic and antimicrobial activities (Vinholes et al., 2015). However, and despite their abundance in diet and credible effects on the prevention of various OS-related diseases, only recently have dietary polyphenols been truly recognised by nutritionists, researchers and food manufacturers. Their preventive effects, in terms of cardiovascular, neurodegenerative diseases, and cancer, have been deduced from epidemiologic data (*in vitro* and *in vivo*) and result in nutritional recommendation (Dai and Mumper, 2010). The most recently identified property of polyphenols is their effect on long-term diabetes complications, including retinopathy, nephropathy, and neuropathy (Bahadoran et al., 2013).

The main phenolic compounds identified in the extracts of *Salvia* and *Plectranthus* are the hydroxycinnamic acids and their derivatives, such as rosmarinic (Rice et al., 2011), chlorogenic (Chassagne and Morgan, 2020)**,** carnosic, and salvianolic acids. Among the most abundant cinnamic acids is caffeic acid (CA) (3,4-dihydroxycinnamic acid) (Pal et al., 2011), described as having a wide variety of biological activities, including antioxidant, antithrombotic, antihypertensive, antifibrotic, antiviral, and antitumour properties (Rajendra Prasad et al., 2011). While the isolation of hydroxycinnamic acids is mostly described in the literature in aqueous extracts, in the case of *P. ecklonii* there have been attempts by scientists to test other extracts, specifically hydroalcoholic extracts. Despite traditional preparations of plant extracts using water (e.g., infusions, decoctions, and poultices) (Rabe and Van Staden, 1997), there are reports of studies in which methanolic extracts have shown a higher content of phenolic compounds when compared to aqueous ones (Krishnaiah et al., 2011). RA (Rice et al., 2011) is an ester of CA (Pal et al., 2011) with 3,4-dihydroxyphenyl lactic acid (Petersen, 2013), the major component of polar extracts from many plants of the *Lamiaceae* family (Falé et al., 2009) and, presumably, one of the main compounds responsible for the potent antioxidant activity of *Lamiaceae* plants (Ozgen et al., 2008). Besides its well-studied antioxidant activity, RA acts as an enzyme inhibitor. It is known to interfere with gene expression and signalling pathways related to cancer prevention and presents antiviral, antibacterial, and anti-inflammatory properties (Bhatt et al., 2013). In 2010, Figueiredo and colleagues pointed out the presence of RA (Rice et al., 2011) in the aqueous extract of *P. ecklonii* as responsible for the antibacterial activity against *Streptococcus* spp. and for the inhibition of the enzyme glycosyltransferase (GTF) (Figueiredo et al., 2010). Furthermore, in 2009, Falé and colleagues also linked the presence of this compound to the observed effects of AChE inhibition and antioxidant activity (Falé et al., 2009). Besides its well-studied antioxidant activity, RA (Rice et al., 2011) acts as an enzyme inhibitor. It is known to interfere with gene expression and signalling pathways related to cancer prevention and presents antiviral, antibacterial, and anti-inflammatory properties (Bhatt et al., 2013). Whereas CA (Pal et al., 2011) and its derivatives are widespread in the *Labiatae* family, RA (Rice et al., 2011) is restricted to the *Nepetoideae* subfamily (Abdel-Mogib et al., 2002). For this reason, RA (Rice et al., 2011) and two other esters of CA (Pal et al., 2011), known as nepetoidin A (Van Jaarsveld, 2006) and nepetoidin B (Nyila et al., 2009) are used as chemotaxonomic markers for the subfamily *Nepetoideae* (Grayer et al., 2003).

### Flavonoids

**FIGURE 4:**
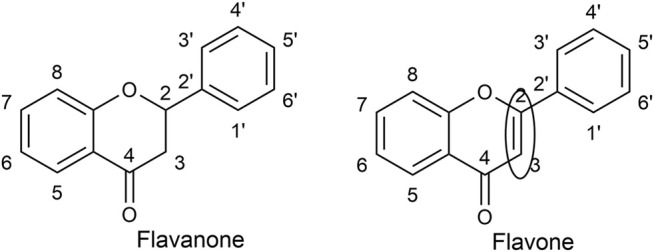
The double bond between C2 and C3 makes it possible to distinguish flavones from flavanones.

Flavonoids are low molecular weight aromatic compounds characterized by a flavanic nucleus and a carbon skeleton with a C6-C3-C6 configuration. Flavonoids contain a skeleton made up of fifteen-carbon atoms, consisting of two benzene rings, joined by a heterocyclic pyrane ring (Kumar and Pandey, 2013). The individual numbering of the flavonoid skeleton is shown in Figure 4 (Martens and Mithöfer, 2005). Flavonoids are well-known for their antioxidant, anti-inflammatory, and cytoprotective activities. Most importantly, they appear in all green plants and constitute a large part of our common daily diet, making them vital components in the prevention of human diseases (Schmidt et al., 2012). Variations in the C ring replacement configurations result in the various subclasses of flavonoids: flavones (e.g., apigenin (Simões et al., 2010) and luteolin (Grayer et al., 2003)), flavanones, isoflavones, flavonols, flavanols (or catechins), and anthocyanidins (Sandhar et al., 2011).

Research reports flavonoids as having many activities (anti-inflammatory, antibacterial, cytotoxic, antitumour, effects on the treatment of neurodegenerative diseases), but the best-described characteristic of the majority of flavonoids is their ability to behave as antioxidants scavenging free radicals and/or chelating metal ions. They are also known to inhibit lipid peroxidation, platelet aggregation, and enzyme activity of COX and LOX enzymes (Asif and Khodadadi, 2013). Flavonoids lacking hydroxyl groups on their B-rings are more active against microorganisms than are those with the -OH groups (Cowan, 1999). Flavones differ from flavanones by the presence of a double bond between C2 and C3 in the heterocyclic flavonoid skeleton. The B ring is connected to C2 and there are usually no C3 substitutes. Flavones occur mainly as 7*-O*-glucosides, although substitution can be found in any other hydroxylated position (Martens and Mithöfer, 2005).

A study with the hydroalcoholic extract of *P. ecklonii* leaf extract showed varying degrees of antimicrobial activity and resulted in the identification of the flavones: vitexin (Gaspar-Marques et al., 2006), isovitexin (Amoah et al., 2016), apigenin 7-*O*-*β*-glucoside (Devappa et al., 2011), apigenin 4',6-dimethoxy-7-*O*-*β*-glucoside (Teixeira et al., 1997), luteolin 7-*O*-glucoside (Kubínová et al., 2013), apigenin (Simões et al., 2010) and luteolin (Grayer et al., 2003) (Hawas et al., 2008). Since then, the flavones cirsimaritin (Nyila et al., 2012), ladanein (Gurlal, 2005)**,** and salvigenin (Van Zyl et al., 2008) have been isolated from *P. ecklonii* (Grayer et al., 2010). Apigenin (Simões et al., 2010) and luteolin (Grayer et al., 2003) are frequently found in several plant species. Apigenin (Simões et al., 2010) (4',5,7-trihydroxyiflavone) has gained particular interest in recent years as a beneficial and health promoting agent due to its low intrinsic toxicity. Plants rich in luteolin (Grayer et al., 2003) (3′,4′,5,7-tetrahydroxyflavone) have been used in TCM for treating various diseases such as hypertension, inflammatory disorders and cancer (Lin et al., 2008). Vitexin (Gaspar-Marques et al., 2006) and isovitexin (Amoah et al., 2016), naturally occurring C-glycosylated derivatives of apigenin (Simões et al., 2010), have been known to possess potent anti-diabetic, anti-Alzheimer’s disease (anti-AD), and anti-inflammatory activities (Choi and Lee, 2009). Plant extracts containing vitexin (Gaspar-Marques et al., 2006) (apigenin-8-*C*-*β*-d-glucopyranoside) are reported to possess anti-inflammatory, and antioxidant activities (Borghi et al., 2013). Phytochemical studies that have been reported investigating *P. ecklonii* also include the isolation of two isomeric ortho-quinones, ecklonoquinones A (Śliwiński et al., 2020) and B (Andrade et al., 2018) (Uchida et al., 1980), twelve flavones (Hawas et al., 2008), as well as salvigenin (Van Zyl et al., 2008), cirsimiratin (Nyila et al., 2012) and the corresponding flavanone, 2(*S*)-4',5-dihydroxy-6,7-dimethoxyflavanone (Costa et al., 2018) (Uchida et al., 1980; Grayer et al., 2003). Flavonoids with a 5-hydroxy-6,7-dimethoxy-type substitution in the A-ring, such as salvigenin (Van Zyl et al., 2008), cirsimaritin (Nyila et al., 2012) and cirsiliol (Uchida et al., 1980) flavones, are considered typical in the Labiateae family (Gaspar-Marques et al., 2006). No reference to any bioactivities exercised by ecklonoquinones A (Śliwiński et al., 2020) and B (Andrade et al., 2018) has been found in the literature and therefore they are not discussed in this review.

**FIGURE 5:**
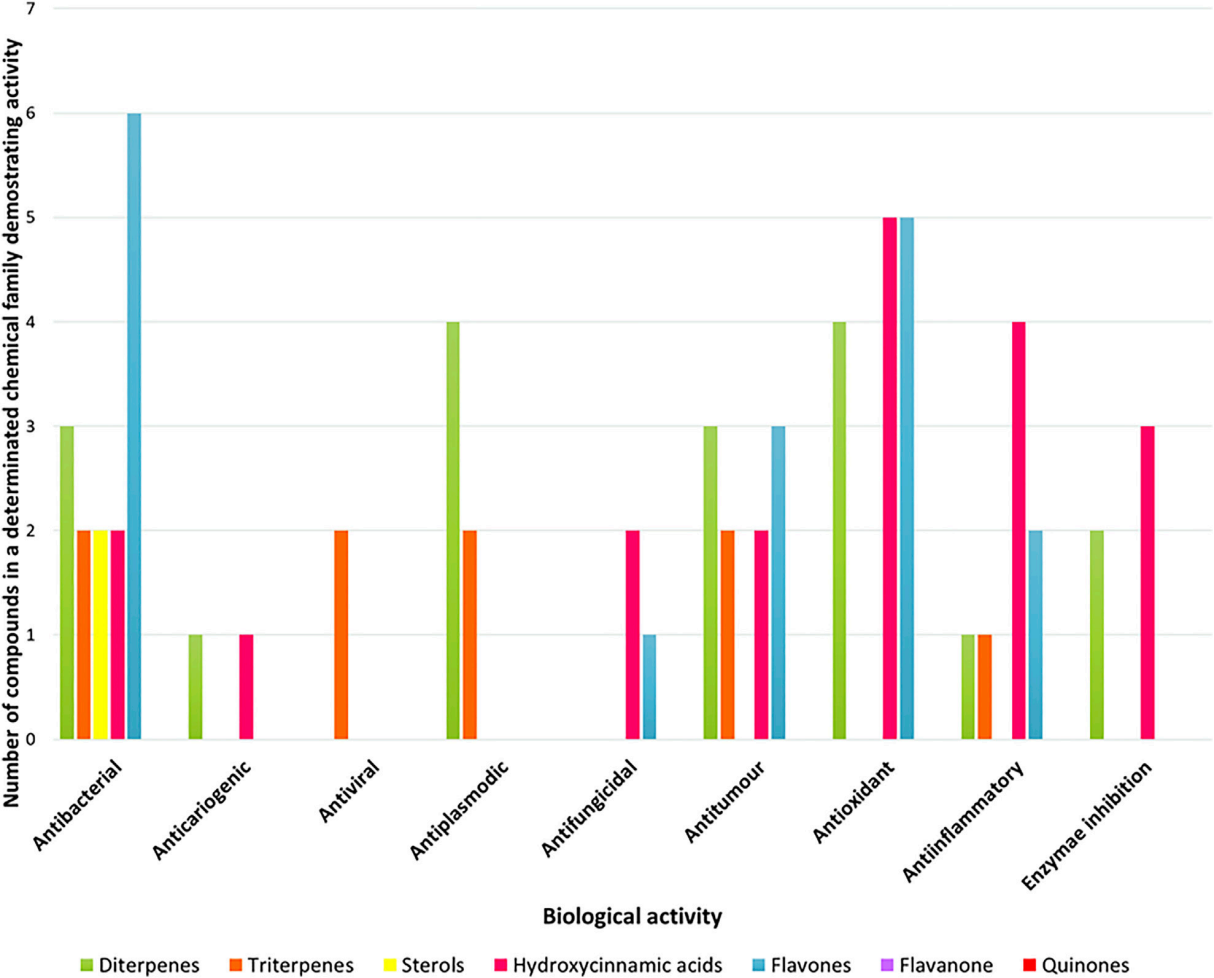
Summary diagram of biological activities demonstrated by each family of compounds.

In the following section, some of the biological activities attributed to the different constituents of *P. ecklonii* will be evaluated and discussed (Figure 5), to try to understand not only its traditional applications, but also the future implications for this plant.

## Biological Activities of Isolated Compounds From *P. ecklonii* Benth

### Antibacterial

The most studied bioactivity in *P. ecklonii* isolated compounds was antimicrobial, such that, different types of microorganisms have been tested. The compounds showed varying degrees of activity against Gram-positive bacteria, Gram-negative bacteria (*Pseudomonas aeruginosa* and *E. coli*), and fungi, such as *Aspergillus niger* and *Candida albicans*. In general, the compounds exert greater antibacterial activity on Gram-positive bacteria (*Staphylococcus*, *Enterococcus*, *Listeria*, and *Streptococcus*).

Parviflorones are natural diterpenes widely distributed among several *Plectranthus* species (Simões et al., 2010).The pigments parviflorone D (Salim et al., 2008) and parviflorone F (Srancikova et al., 2013) were isolated for the first time from an ethereal extract of *Plectranthus parviflorus* (Rüedi and Eugster, 1978). Since then, parviflorone D (Salim et al., 2008) [2α-(4-hydroxy)benzoyloxy-11-hydroxy-5,7,9(11),13-abietatetraen-12-one], has been isolated from *P. strigosus* Benth. (Gaspar-Marques et al., 2008) and *P. ecklonii* Benth. and reported antibacterial activity, including against methicillin- and vancomycin-resistant strains (Simões et al., 2010). Parviflorone F (Srancikova et al., 2013) [11-hydroxy-2α-(3,4-dihydroxybenzoyloxy)-abieta5,7,9(11),13-tetraene-12-one] was also isolated from *P. ecklonii* and *Plectranthus nummularius* Briq. (Narukawa et al., 2001), as well as parviflorone E (Abdel-Mogib et al., 2002) [11-hydroxy-19-(3,4-dihydroxybenzoiloxy)-abieta-5,7,9(11),13-tetraene-12-one] (Figueiredo et al., 2014).

The leaves of members of the *Lamiaceae* family are known to contain terpenoids with antifungal, antibacterial, and insect repellent activities (Cole, 1994). Extracts obtained from the leaves of some *Plectranthus* species in South Africa have shown antibacterial activity (Rabe and Van Staden, 1997). Abietane diterpenes isolated from *Plectranthus elegans* inhibited the growth of Gram-positive bacteria *Bacillus subtilis* (Dellar et al., 1996). The diterpenes isolated from *Plectranthus grandidentatus* and *Plectranthus hereroensis* also proved to be active against resistant Gram-positive bacteria, *Enterococcus faecalis* vancomycin-resistant (VRE) and Methicillin-resistant *Staphylococcus aureus* (MRSA) (Gibbons, 2004; Gaspar-Marques et al., 2006). Concerning ethyl acetate extracts of *P. ecklonii*, two known abietanes, parviflorone D (Salim et al., 2008) and parviflorone F (Srancikova et al., 2013), were isolated and both compounds demonstrated effective activity against *Listeria monocytogenes* (Nyila et al., 2009). The traditional use of *P. ecklonii* for the treatment of gastrointestinal disorders may also be related to its activity against *E. coli* (Nyila et al., 2012), although further studies are needed to support this hypothesis.

**FIGURE 6:**
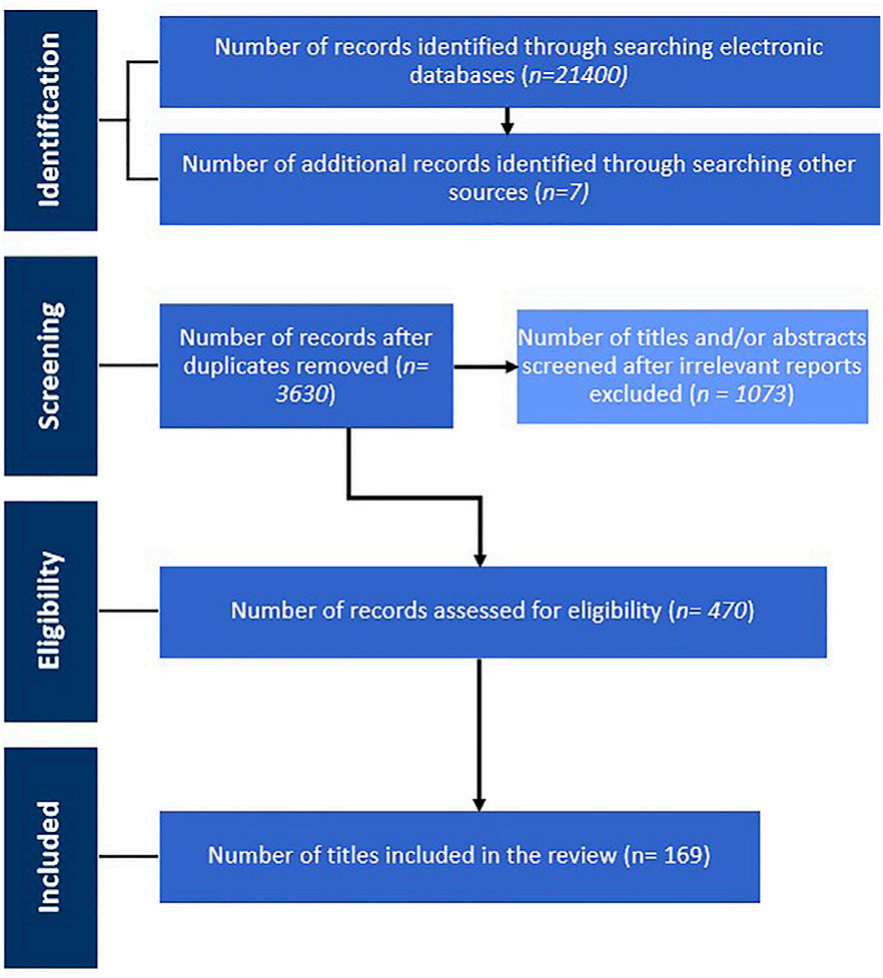
PRISMA flow chart demonstrating the selection process and criteria.

Abietanes parviflorone D (Salim et al., 2008) and F (Srancikova et al., 2013) were also active against *Mycobacterium smegmatis*, *P. aeruginosa*, and *E. faecalis* (Nyila et al., 2009). The antibacterial activity of sugiol (de Albuquerque et al., 2007) was also tested, although authors reported very low activity against Gram-positive *E. faecalis* bacteria (Simões et al., 2010). The leaves of the plant are used for respiratory symptoms, chest pain, and coughing (problems related to tuberculosis), which may be due to the inhibitory growth activity of *M. tuberculosis* presented by parviflorones D (Salim et al., 2008) and F (Srancikova et al., 2013) (Nyila et al., 2009). Parviflorone D (Salim et al., 2008) has also inhibited the growth of *S. aureus* (Nyila et al., 2009; Simões et al., 2010), which possibly justifies the use of the aerial parts of the plant in Zimbabwe for skin diseases and hyperpigmentation problems (Lukhoba et al., 2006). Antibacterial activity of parviflorone D (Salim et al., 2008) has been reported against *Staphylococcus* and *Enterococcus* species, including against MRSA and VRE strains (Simões et al., 2010). Even the rearranged abietane 2ß-(4-hydroxy)benzoyloxy (Figure 6) obtained in 2010 by Simões and colleagues from parviflorone D (Salim et al., 2008) showed antibacterial activity against some *Staphylococcus* and *Enterococcus* strains when tested against Gram-negative and Gram-positive bacteria (Simões et al., 2010).

According to Cowan, the mechanism responsible for the antibacterial activity of diterpenes may be associated with the breakdown of the bacterial membrane by lipophilic compounds (Cowan, 1999).

The high cost of synthetic drugs and the problem of multidrug resistance has increased the need to exploit the anti-Listeria potential of medicinal plants. Plant extracts are affordable and accessible, which has led to the use of medicinal plants as an alternative in the treatment of listeriosis. *P. ecklonii* Benth. is one of the plants traditionally used to treat the symptoms associated with listeriosis infection (Lukhoba et al., 2006). Many organisms, including the opportunistic pathogen *Listeria monocytogenes*, appear more often as biofilms, such as in healthcare-acquired “hospital” infections. An ethyl acetate extract from *P. ecklonii* showed anti-*Listeria* activity with a minimum inhibitory concentration (MIC) of 0.5 mg/ml. Parviflorone D (Salim et al., 2008) and F (Srancikova et al., 2013) showed even higher activity in the breakdown of *L. monocytogenes* biofilm with a MIC of 15.6 μg/ml and 31.25 μg/ml, respectively (Table 2) (Nyila, 2010). Although the results illustrate a possible use of the compounds as disinfection agents, further studies should be carried out to investigate their potential for effectively removing *Listeria* biofilm from contaminated surfaces.

**TABLE 2:** MICs and IC_50_ values of the compounds Parviflorone D, Parviflorone F and Sugiol against different tested microorganisms.

Microorganism	Parviflorone D Salim et al. (2008)		Parviflorone F Srancikova et al. (2013)		Sugiol de Albuquerque et al. (2007)		Ref.
	MIC (μg/ml)	IC50 (μM)	MIC (μg/ml)	IC50 (μM)	MIC (μg/ml)	IC50 (μM)	
*S. aureus* ATCC 43866	15.62	-	-	-	-	-	Simões et al. (2010)
*S. aureus* CIP 106760	15.62						
*E. faecalis* ATCC 51299	7.81				62.5		
*E. faecalis* FFHB	3.90				-		
*M. smegmatis*	39.06		39.06				Nyila et al. (2009)
*M. tuberculosis*	190		95				
*L. monocytogenes*	15.6		31.25				
*E. coli*	31.25		31.25				Nyila, (2010)
*P. aeruginosa*	31.25		31.25				
*P. falciparum*	-	5.3		3.11		1.4–3.4	Van Zyl et al. (2008), Bero et al. (2009)

S, Staphylococcus; E. faecalis, *Enterococcus faecalis*; M, Mycobacterium; L, Listeria; *E. coli*, *Escherichia coli*; *P. aeruginosa*, *Pseudomonas aeruginosa*; P. falciparum, Plasmodium falciparum; ATCC, American Type Culture Collection; MIC, Minimal Inhibitory Concentration; IC_50_, Half maximal inhibitory concentration; Ref, Reference(s)

Different reports have shown that UA (Lukhoba et al., 2006) and OA (Dellar et al., 1996) exhibit antimycotic, antitumoral, antibacterial, antiviral, and antiparasitic properties. UA (Lukhoba et al., 2006) and OA (Dellar et al., 1996) present remarkable antimicrobial activities and they act against important human pathogens, such as mycobacteria, HIV, and different protozoal species (Jesus et al., 2015). UA (Lukhoba et al., 2006) and its derivatives have been shown to possess antimicrobial activity, for example, as inhibitors of Gram-positive *S. aureus*, Gram-negative organisms (*P. aeruginosa* and *E. coli*), and *Microsporium lenosum* (Zaletova et al., 1986). OA (Dellar et al., 1996) showed antimicrobial activity against *Bacillus subtilis*, methicillin-sensitive *S. aureus* (MSSA), and MRSA (Sun et al., 2006). When used against *M. tuberculosis*, both OA (Dellar et al., 1996) and UA (Lukhoba et al., 2006) presented anti-tuberculosis potential (Jiménez-Arellanes et al., 2007). In 2010, Figueiredo and colleagues pointed out that the presence of RA (Rice et al., 2011) in the aqueous extract of *P. ecklonii* is responsible for the antibacterial activity against *Streptococcus* spp. (Falé et al., 2009).

### Anticariogenic

Dental caries has been the oral pathology most responsible for the loss of tooth structure with *Streptococcus mutans* being considered the main cause of this dental disease. Despite the diversity of human oral flora composition, two *Streptococci* strains, *Streptococcus mutans* and *Streptococcus sobrinus*, have been implicated as the primary etiologic agents of dental caries (Hamada and Slade, 1980; Song et al., 2006; Bernardes et al., 2010). One of the most important virulence factors of these species is their ability to produce glucosyltransferases (GTFs) and multiple glucan-binding proteins (Gbp proteins), which are thought to promote adhesion of bacteria to the tooth surface, contributing to the formation of dental plaque (Song et al., 2006; Matsumoto-Nakano, 2018). For biofilm formation, *S. sobrinus* and *S. mutans* must have the ability to adhere to a surface. Therefore, if compounds make such adherence impossible, both the biofilm formation process and its subsistence will be compromised.

**FIGURE 7:**
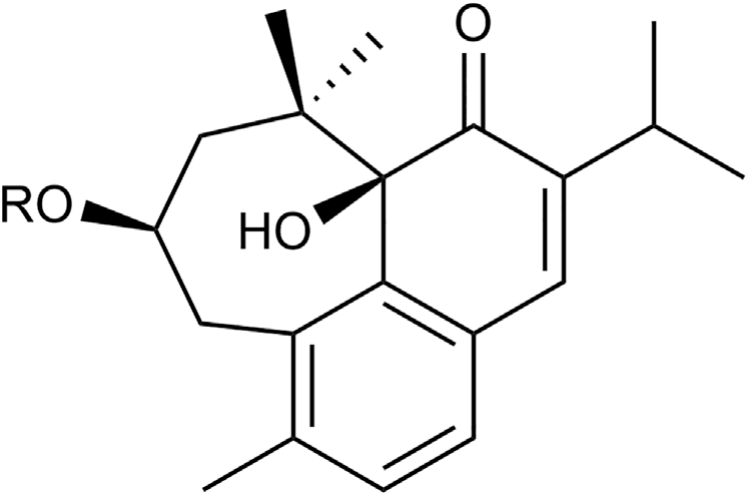
Abietane 2ß-(4-hydroxy)benzoyloxy.

The aqueous extract of *P. ecklonii* has been reported to have antibacterial activity against *S. mutans* and *S. sobrinus* and inhibited the enzyme GTF. The main compound present in *P. ecklonii* said to be responsible for this action is RA (Rice et al., 2011), however, the authors have noted that the inhibitory effect of the acid on biofilm formation did not differ significantly from the effect observed for the aqueous extract (Figueiredo et al., 2010). The methanol extract from *P. ecklonii* leaves revealed the presence of parviflorone E (Narukawa et al., 2001)**,** together with RA (Rice et al., 2011), resulted in higher anti-cariogenic activity (Figueiredo et al., 2014), confirming this species importance in the prevention of oral diseases. Furthermore, the antimicrobial activity of an aqueous extract of *P. ecklonii,* containing RA (Rice et al., 2011), showed the extract as being active in bacteria, particularly against Gram-positive *S. epidermidis*, normally found in skin flora, justifying its traditional use and demonstrating its potential for skin application (Nicolai et al., 2020). By contrast, in an *in vitro* study of the antimicrobial activity of hydroalcoholic extracts (EtOH/H_2_O) of *Rosmarinus officinalis* against *S. mutans*, *S. salivarius*, *S. sobrinus*, *S. mitis*, *S. sanguinis*, and *E. faecalis*, neither RA (Rice et al., 2011) nor the two ester derivatives prepared from it showed antimicrobial activity against the selected microorganisms (Bernardes et al., 2010). Further studies should therefore be carried out to confirm the true action of the compound (Rice et al., 2011) against the species *Streptococcus* concerned.

### Antiviral

Among the various important pharmacological properties attributed to OA (Dellar et al., 1996) is its hepatoprotective effect. It has been shown that OA (Dellar et al., 1996) is not only effective in protecting the liver from acute chemically induced liver injury but also protects the liver from fibrosis and cirrhosis caused by chronic liver diseases (Poolier and Goossens, 2012). OA (Dellar et al., 1996) has been marketed in China as a human over-the-counter (OTC) drug for the treatment of liver diseases such as acute and chronic hepatitis and a recent report shows that an extract, containing both acids (Lukhoba et al., 2006) and (Dellar et al., 1996), has significantly suppressed the replication of the hepatitis C virus (Kong et al., 2013). Given the anti-viral potential of these compounds, the authors propose the inclusion of these two compounds in clinical trials as monotherapy or combination with other hepatitis C antivirals. Furthermore, considering the extensive antiviral activites shown by *P. ecklonii*, it could be interesting to further investigate the effect of its active compounds for the treatment of other common viral infections, such as, Herpes simplex virus (HSV) and Hand-foot-and-mouth disease (caused by the coxsackievirus virus).

### Antiplasmodic

Malaria is currently one of the world’s public health concerns due to factors such as resistance to chemotherapy, poor hygiene conditions, poorly managed vector control programs, and lack of approved vaccines. There has been a general call for the use of natural products (NPs) as medicines or as a basis for the development of new antimalarials, to avoid the problems related to drug resistance (Amoa Onguéné et al., 2013). Of the four types of parasite associated with human malaria, *Plasmodium falciparum* is responsible for the most severe cases and is therefore used in most studies assessing compound activity in these species (Bero et al., 2009).

The antimalarial properties of *Plectranthus* species were determined by Van Zyl and colleagues in 2008; seven abietane diterpenes, including parviflorones D (Salim et al., 2008), F (Srancikova et al., 2013), and E (Abdel-Mogib et al., 2002), were isolated and their antiplasmodial activity and ability to inhibit the formation of β-haematin were tested (Van Zyl et al., 2008). Parviflorones D (Salim et al., 2008) and F (Srancikova et al., 2013) were isolated from *P. ecklonii* leaves and exhibited antiplasmodial activity (van Zyl et al., 2007). The lipophilic nature of abietane diterpenes allows them to easily cross erythrocyte and parasitic membranes to accumulate in the parasite vacuole. It is believed that the inhibitory effect of these compounds is related to their ability to inhibit the formation of β-haematin. This is an important effect since the malaria parasite degrades haemoglobin and the released haem, which is toxic to the parasite, is normally converted to the inert malaria pigment, β-haematin. Parviflorone F (Srancikova et al., 2013) was more effective than quinine and 62% as active as chloroquine, two conventional antimalarials. Parviflorone E (Abdel-Mogib et al., 2002), isolated from *P. purpuratus* (subspecies tongaensis) (compound also existing in *P. ecklonii*), has also been shown to be more active than quinine. When combined with quinine, the compounds Parviflorone F (Srancikova et al., 2013) and E (Abdel-Mogib et al., 2002) interacted in an additive manner (Van Zyl et al., 2008). With (Srancikova et al., 2013) and (Abdel-Mogib et al., 2002) showing higher efficacy than quinine in treating malaria, and the fact that *P. ecklonii* grows in Africa where other parasitic diseases exist, studies on other parasitic diseases should be performed, for example, sleeping sickness produced by *Trypanosoma brucei rhodesiense* and *Trypanosoma brucei gambiense*. Furthermore, it would be appropriate to suggest investigation of these compounds on other diseases also treated by quinine. Most diterpenes are known to combine high antiprotozoal activity with high toxicity to mammalian cells (e.g., kidney epithelial cells), hepatoma cells, and colon carcinoma cells. The cytotoxic profile of these compounds indicated a low degree of specificity towards the malaria parasite, making them weak candidates for the development of antimalarial agents. However, the authors suggested that further chemical modifications of these naturally-derived compounds and analogues of Parviflorone F (Srancikova et al., 2013) could result in more active antiprotozoal agents with decreased toxicity (Van Zyl et al., 2008). According to Bero and colleagues, the diterpene sugiol (de Albuquerque et al., 2007) is also a promising antimalarial agent with half-maximal inhibitory concentration (IC_50_) between 1.4 and 3.4 μM, determined *in vitro* on *P. falciparum* strains (Bero et al., 2009). Combinations of compounds (Srancikova et al., 2013), (Abdel-Mogib et al., 2002) and (de Albuquerque et al., 2007), should be carried out in specific formulations to identify any additive properties.

Several studies have demonstrated a growth inhibitory effect of flavonoids, in particular flavonol quercetin and flavone luteolin (Grayer et al., 2003), in protozoa of the genera *Toxoplasma*, *Trypanosoma* and *Leishmania*. Most studies involve malaria and flavonoids isolated by biologic studies of species used in traditional medicine (Lehane and Saliba, 2008). The *in vitro* antiplasmodial activity of eleven flavonoids, including the flavones apigenin (Simões et al., 2010) and luteolin (Grayer et al., 2003), has been tested against a chloroquine sensitive strain (3D7) and a chloroquine resistant strain (7G8) of *P. falciparum*. The most active compound against both strains was luteolin (Grayer et al., 2003), with IC_50_ values of 11 ± 1 μM and 12 ± 1 µM for the 3D7 and 7G8 strains, respectively. It was also found that luteolin (Grayer et al., 2003) prevents the parasite’s growth progression beyond the trophotozoic phase and does not affect the parasite’s susceptibility to chloroquine or artemisinin antimalarial drugs. The combination of low concentrations of different flavonoids appears to produce an additive antiplasmodic effect (Lehane and Saliba, 2008). When isolated from *P. strigosus*, the flavone salvigenin (Van Zyl et al., 2008) showed low activity against *P. aeruginosa* (Gaspar-Marques et al., 2006). It also proved to be a very weak inhibitor of *S. aureus*, as opposed to apigenin (Simões et al., 2010), which was active in MSSA and MRSA-type strains (MIC 3,9–15,6 µg/ml) (Sato et al., 2000).

Acids (Lukhoba et al., 2006) and (Dellar et al., 1996) have also been described as potent agents against *Leishmania* species. These triterpenic acids are active against amastigotes (IC_50_ 7–120 nM) and display moderate activity in the promastigotes (IC_50_ 51-137 nM) of *Leishmania donovani* and *L. major* (Tan et al., 2002). To establish anti-Leishmania SAR, in 2011, Peixoto and colleagues prepared OA (Dellar et al., 1996) derivatives and compared their IC_50_ values (Peixoto et al., 2011). The results of this *in vitro* study suggested that an increase in lipophilicity in the carbon 17 (C17) is more relevant to anti-*Leishmania* activity than an increase in lipophilicity in C3.

### Anti-fungicidal

Dichloromethane extracts of *P. ecklonii* were screened for antibacterial and antifungal activities using the agar well and trench diffusion methods. Although both methods produced inconsistent results, high biological activity was observed when *P. ecklonii* was tested against *Candida* species by the trench diffusion technique (Gurlal, 2005). Abietane diterpenes isolated from *Plectranthus elegans* inhibited spore germination of the fungus *Cladosporium cucumerinum* (Dellar et al., 1996). Anti-fungicidal activity of the rearranged abietane 2ß-(4-hydroxy)benzoyloxy (Figure 7), obtained by Simões and colleagues in 2010, from parviflorone D (Salim et al., 2008)**,** showed promising results against *C. albicans* (Simões et al., 2010). The flavone salvigenin (Van Zyl et al., 2008), isolated from *P. strigosus,* showed low activity against *C. albicans* (Gaspar-Marques et al., 2006). Antifungal activity against *Aspergillus niger* has also been reported for the compounds nepetoidin A (Van Jaarsveld, 2006) and nepetoidin B (Nyila et al., 2009) (Grayer et al., 2003). Nepetoidin B has also shown activity against *Cladosporium herbarum*. (Figueiredo et al., 2010).

### Antitumour

Abietane diterpenes display an array of biological activities including cytotoxic and antiproliferative activities against human tumour cells (Burmistrova et al., 2013). Abietane diterpenes, especially those containing quinone moieties, deserve greater attention because several cancer chemotherapeutic agents also possess the quinone structural feature (Fronza et al., 2012). Biological membranes are potential targets of abietane diterpenes due to their lipophilic character. Studies show that cell death induced by these compounds may not follow a single mechanism, but rather several ones. It is also possible that the structural properties of diterpenes can influence or determine their molecular mode of cell death (Spiridonov et al., 2003; Fronza et al., 2012).

Sugiol (de Albuquerque et al., 2007) was reported to exhibit modest growth inhibitory activity against human breast, lung, and colon cancer cell lines (Son et al., 2005). In a study involving human pancreatic cancer cell line MIA PaCa-2, sugiol (de Albuquerque et al., 2007) influenced the relaxation activity of human DNA topoisomerases I and II. This compound showed preferential inhibition of topoisomerase I (IC_50_ of 2.8 µM) and demonstrated lower IC_50_ values than camptothecin, a classical topoisomerase I inhibitor (28.0 µM) (Fronza et al., 2012).

Recently, the anticancer effect of parviflorone D (Salim et al., 2008) was also evaluated in human breast cancer cells (Costa et al., 2018) and the results indicated further studies should be done towards a potentially therapeutic application. Furthermore, since parviflorone D (Salim et al., 2008) demonstrates limited water solubility, the formulation of parviflorone D (Salim et al., 2008) into hybrid nanoparticles to assist in longer-term drug delivery and therapeutic effect has been documented. It was reported that parviflorone D (Salim et al., 2008) showed cytotoxic activity towards human melanoma cells (A375), human ‘normal-like’ fibroblasts (Detroit 551 cell line), and mouse cell lines (B16V5). Further to this, α-MSH-conjugated hyaluronic and oleic acid-coated nanoparticles were formulated and showed promising results as long-term drug-release platforms in the targeted and localized therapeutic action towards melanoma cell lines (Silva et al., 2016). Additionally, studies investigating the use of optimized nanosystems for parviflorone D (Salim et al., 2008) delivery to pancreatic tumour cells, using erlotinib nanoparticles conjugated to parviflorone D **(1**) loaded albumin nanoparticles showed promising delivery to the tumour site and high antiproliferative effect in BxPC3 cell lines (Santos-Rebelo et al., 2018; Santos-Rebelo et al., 2019). During a study into the *in vitro* anti-inflammatory activity of *Plectranthus* NPs, parviflorone D (Salim et al., 2008), along with royleanone isolated from *P. grandidentatus*, demonstrated cytotoxic activity two times greater than the compound with the lowest viability. This cytotoxic evaluation showed parviflorone D (Salim et al., 2008) as having high toxicity for RAW 264.7 cells (Andrade et al., 2018). Parviflorone D (Salim et al., 2008) isolated from *P. ecklonii* showed cytotoxicity against human leukaemia cell lines CCRF-CEM and lung adenocarcinoma cell lines A549, by inducing apoptosis and influencing ROS levels (Śliwiński et al., 2020). In another recent study, parviflorone D (Salim et al., 2008) induced apoptosis in a human H7PX glioma cell line, obtained from brain tumour glioblastoma multiforme cells and demonstrated the highest amount of cytotoxicity against CCRF-CEM and A459 cell lines, when compared to other royleanone abietane diterpenes. Parviflorone D (Salim et al., 2008) produced 73% of early and late apoptosis when compared to untreated cells. The authors suggest that the high levels of phosphorylated histone in the H7PX cell lines, indicative of double-strand breaks, a decrease in the mitochondrial membrane potential and a change in pro and anti-apoptotic gene expression all contributed to apoptosis (Śliwiński et al., 2020). Furthermore, a study into the *in vitro* bioactivity of parviflorone D (Salim et al., 2008) highlighted the different pathways involved in the cytotoxic activity of the compound against multiple human cancer cell lines, including HL-60, U-937, MOLT-3, and K-562. The apoptosis induced by parviflorone D (Salim et al., 2008) was also attributed to the reduction in the mitochondrial membrane potential and influencing the levels of ROS. However, also, the inhibition of extracellular signal-regulated kinases (ERKs) enhanced tumorous cell death (Burmistrova et al., 2015). These results, along with those previously listed indicate parviflorone D (Salim et al., 2008) as having huge potential as a chemotherapeutic drug. Protein kinases C (PKC), which are involved in a variety of carcinogenic processes, have become a popular target for cancer therapy over the years. By using molecular docking studies, it has been possible to predict the enhanced activity of derivatized royleanones in cancer cell lines. Parviflorone D (Salim et al., 2008) showed activity against aggressive breast cancer cells, such as SUM159 sphere stem cells, as well as inhibiting MCF-7, SkBr3, and SUM159 cell lines, but also demonstrated a large interaction profile when binding sites were substituted with different moieties. Parviflorone D (Salim et al., 2008) PKC isoforms demonstrated the highest interaction profile when compared to other diterpene isoforms studied (Isca et al., 2020). Triple-negative breast cancer (TNBC), a rare and more aggressive cancer, in which the tests for estrogen receptors, progesterone receptors, and excess HER2 protein come back negative, has been studied with parviflorone D (Salim et al., 2008) to assess the therapeutic action of (Salim et al., 2008) in a model of TNBP, MDA-MB-231 cell lines. Overall, it was reported that (Salim et al., 2008) reduced the cell mobility and chemotactic invasion and induced apoptosis, once again demonstrating the potential of Parviflorone D (Salim et al., 2008) in chemotherapeutic drugs (Saraiva et al., 2020).

As opposed to parviflorone D (Salim et al., 2008), parviflorone F (Srancikova et al., 2013) has been shown to induce cell death by avoiding the mitochondrial permeability and initiating an alternative pathway that does not involve inhibiting anti-apoptotic proteins Bcl-2 and Bcl-X_L_. Parviflorone F (Srancikova et al., 2013) showed anti-proliferative activity ranging from IC_50_ values of 4.49 - 4.99 μM, across a variety of human cell lines, including TNBC MDA-MB231, breast cancer MCF-7 and lung carcinoma A549. It has been suggested that the oxidation level of the abietane ring affects the antiproliferative selectivity of the compound. When compared to parviflorone D (Salim et al., 2008), parviflorone F (Srancikova et al., 2013) demonstrated higher cytotoxicity in Vero cell lines (Garcia et al., 2019).

Since *P. ecklonii* is a common species of South Africa, antitumour drug development using isolated compounds (Salim et al., 2008) and (Srancikova et al., 2013) could be of importance for countries with less access to other resources. When compared to the preparation of the common anticancer treatment paclitaxel, extracting parviflorones D (Salim et al., 2008) and F (Srancikova et al., 2013) from the aerial parts of *P. ecklonii* could be more accessible than, for example, from the bark of the Pacific yew tree (*Taxus brevifolia*). *P. ecklonii* is a source of different bioactive compounds, not just one of key interest, as in the case of *T. brevifolia*, therefore, in terms of economising and sustainable use of resources from natural products, *P. ecklonni* could be a legitamate alternative. Furthermore, with parviflorones D (Salim et al., 2008) and F (Srancikova et al., 2013) being recorded as demonstarting even higher antitumour activity than standard antitumour agents, the appliaction of using these compounds for *in vitro* investigation standards could be considered. The diterpene sugiol (de Albuquerque et al., 2007) demonstrated preferential inhibition of topoisomerase 1, with an IC_50_ value of 2.8 µM, lower than that of camptothecin (28.0 µM) (Fronza et al., 2012). Reported adverse effects of camptothecin have reduced it’s clinical use, providing the oppourtity for alternative drug leads. Given its recorded potency, (de Albuquerque et al., 2007) should be further investigated for use in cancer therapy. Compounds, such as abietane diterpenes, could be studied in combination with current clinical drugs, to improve their activity, overcome resistances or mitigate and/or prevent adverse effects. All these results suggests potential therapeutic properties for Parvifloron D (Salim et al., 2008), specially with the help of the nanotechnology to enhance its solubility.With parviflorones D (Salim et al., 2008) and F (Srancikova et al., 2013) being recorded as demonstrating even higher antitumour activity than standard antitumour agents, the appliaction of these compounds for *in vitro* standard studies could be considered, as well as in combination with current clinical drugs, to improve their activity, overcome resistances or mitigate and/or prevent adverse effects.

The antitumour activity and multifunctionality of triterpenoids is attributed to different mechanisms, including, inhibiting NF-κB and topoisomerases activation, inducing an apoptotic response, blocking signal transducer and activating angiogenesis and transcription (D’yakonov et al., 2017). The use of triterpenic compounds, such as UA (Lukhoba et al., 2006) and OA (Dellar et al., 1996), has long been recommended in Japan as a skin cancer therapy (Muto et al., 1990) since both acids have effectively inhibited the promotion and initiation of skin tumours in rats. Cosmetic preparations containing one or both acids are even patented in Japan for topical preventive use of skin cancer (Liu, 1995). There is at least one patented pharmaceutical preparation containing OA (Dellar et al., 1996) for the treatment of non-lymphatic leukaemia (granulocytic and monocytic) without adverse side effects (Liu, 1986). Several studies have indicated that UA (Lukhoba et al., 2006) and its derivatives inhibit the growth of cancer cells by interrupting the cell cycle and stimulating apoptosis (Liu, 2005). In HT-29 colon cancer cells, UA (Lukhoba et al., 2006) decreased cell proliferation in a dose- and time-dependent manner, suggesting that it may be a potent agent for the treatment of colorectal cancer (Andersson et al., 2003; Shan et al., 2009). Another study suggests UA (Lukhoba et al., 2006) as a potential chemopreventive agent in metastatic breast cancer (Yoeh et al., 2010). Cancer is a multifactorial disease, with multiple symptoms and targets; interest in drugs possessing multiple biological actions, such as antitumour and anti-inflammatory, are of increasing interest for their combinations of action, rather than single modes of action. As an example, COX-2 and Leukotrienes (LTs) are involved in the inflammatory process, which have also been linked to the mechanisms of action involved in cancer. In colon cancer HT-29 cells, antitumour effects of RA (Rice et al., 2011) have been related to its ability to inhibit COX-2 activation by AP-1 inducing agents (Hossan et al., 2014). LTs are significantly involved in the immunoregulation process of various inflammatory-dependent diseases, including asthma, and various allergic conditions They are initially biosynthesized by 5-LOX from arachidonic acid. CA (Pal et al., 2011) has been shown to have anti-inflammatory properties as a selective inhibitor of 5-LOX and thus of LT biosynthesis (Yasuko et al., 1984). CA (Pal et al., 2011) also inhibits PKC (Gamaro et al., 2011) and the activation of NF-kB, induced by ceramides in human myeloid leukaemia cell line U937 (Nardini et al., 2001). CA (Pal et al., 2011) was found to diminish NO and prostaglandin E2 (PGE2) production in LPS-stimulated RAW264.7 cells. Additionally, mRNA levels of TNF-*α*, COX-2, and iNOS were downregulated by CA (Pal et al., 2011) (Yang et al., 2013).

Due to the multiple biological activities of flavonoids (anti-inflammatory, antioxidant, antiproliferative, and antibacterial), there have been many studies towards their application as anti-tumour and radiosensitizing agents. For example, cirsiliol (Uchida et al., 1980) has been investigated as a possible radiosensitizer in non-small cell lung cancer (NSCLC) (Kang et al., 2013). Most lung cancer patients are diagnosed at an advanced and inoperable stage, with radiotherapy being their only effective treatment option. Unfortunately, radioresistance of tumours remains a critical obstacle (Provencio et al., 2010). Results show that cirsiliol (Uchida et al., 1980) reduces the proliferation of NSCLC by inhibiting the expression (but not activation) of the Notch-1 gene (Kang et al., 2013).

Several studies have shown that many flavonoids, including luteolin (Grayer et al., 2003) and apigenin (Simões et al., 2010), inhibit the proliferation of various normal and tumoral cells, derived from almost all tissues (Packer et al., 2004). Apigenin (Simões et al., 2010) is a powerful inhibitor of cell proliferation and angiogenesis in human endothelial cells. It inhibits the expression of vascular endothelial growth factor (VEGF) via alpha-1 hypoxia inducing factor degradation (HIF-1α) (Osada et al., 2004) and the growth of human cervical carcinoma HeLa cells and neuroblastoma cell lines, a paediatric tumour (Zheng et al., 2005). Apoptosis of HeLa cells by inducing p53 gene expression suggests the potential of apigenin (Simões et al., 2010) in the development of a preventive agent for cervical cancer. Another study confirmed this chemopreventive action of apigenin (Simões et al., 2010), this time in the treatment of pancreatic cancer, by the inhibition of NF-κB activation (Wu et al., 2014). Although it does not appear so, the anti-proliferative cell activity of flavonoids is specific, depending on the type of cell and the structure of the flavonoid. For example, neither apigenin (Simões et al., 2010) nor luteolin (Grayer et al., 2003) shows the significant growth-inhibiting activity of 4A5 cells in melanoma B16 (Packer et al., 2004).

### Antioxidant

The current modern-day lifestyle causes excessive free radical production and reactive oxygen and/or nitrogen species (ROS/RNS). Antioxidants are defined as compounds that can delay, inhibit, or prevent the oxidation of oxidizable materials by scavenging free radicals and diminishing oxidative stress (OS) (Dai and Mumper, 2010). The production of free radicals is common place during normal aerobic cellular metabolism and can perform various functions as signalling and provide protection against infections (Sharma et al., 2012). However, free radical overproduction results in OS, a detrimental process that can cause oxidative damage of different biomolecules (such as enzymes, proteins, lipids, and nucleic acids) inhibiting their normal function and causing many diseases (Valko et al., 2007). OS has been implicated in the development of chronic degenerative diseases, including cardiovascular and respiratory diseases, neurodegenerative disorders (Alzheimer’s disease (AD) and Parkinson’s disease (PD)), *diabetes mellitus*, rheumatoid arthritis, and different types of cancer, as well as in the aging process (Phaniendra et al., 2015), discovering natural compounds with good scavenging capacity against ROS imperative.

Plant antioxidants are composed of a broad variety of different substances like ascorbic acid (vitamin C) and tocopherols, polyphenolic compounds, or terpenoids (Graßmann, 2005). Evidence of terpene antioxidant behaviour comes from the increasing number of publications published in recent years, focusing on their source, structures, and mechanisms, through which they exert their pharmacological and possible therapeutic activities (Gonzalez-Burgos and Gomez-Serranillos, 2012). One of the most frequently employed methods used to detect the presence of antioxidant compounds is the 2,2-Diphenyl-1-picrylhydrazyl (DPPH•) radical scavenging assay (Akar et al., 2017). In 2005, Chao and colleagues reported that although sugiol (de Albuquerque et al., 2007) had low inhibitory activity against DPPH radical, it could effectively reduce intracellular production of ROS in lipopolysaccharide (LPS) stimulated macrophages (Chao et al., 2005). When compared to the standard compound, ascorbic acid, sugiol (de Albuquerque et al., 2007) showed significant scavenging activities of DPPH, nitric oxide (NO), superoxide, and hydroxyl free radicals in a concentration-dependent manner (Bajpai et al., 2014). Besides, sugiol (de Albuquerque et al., 2007) showed an inhibitory effect of lipid peroxidation of 76.5% compared with α-tocopherol (80.13%) and butylated hydroxyanisole (BHA) (76.5%), two well-known synthetic antioxidants. However, increasing concern concerning these synthetic antioxidants in promoting liver damage and carcinogenic processes merits the search for alternative antioxidant sources, such as, from *Plectranthus* spp. Another diterpene isolated from *P. ecklonii* which demonstrated dose-dependent anti-radical activity was parviflorone D (Salim et al., 2008). This compound had antioxidant properties equivalent to hydroxyl butyltoluene (BHT), but lower than quercetin, two other synthetic antioxidants (Rijo et al., 2009). The antioxidant activity of parviflorone F (Srancikova et al., 2013) and E (Abdel-Mogib et al., 2002)**,** isolated from the leaves of *P. nummularius* Briq., was also evaluated by the DPPH method. Both compounds showed a higher uptake capacity of the DPPH radical than that of the α-tocopherol (Narukawa et al., 2001). It is most probable that the quinone moiety present in the abietane diterpenes, such as in parviflorone D (Salim et al., 2008), aids in stabilizing free radicals.

Phenolic acids, tannins and flavonoid compounds, which are subgroups of phenolics, are known to be potent antioxidants (Ramu et al., 2012). Studies demonstrate a positive and highly significant relationship between total phenolics and antioxidant activity (Velioglu et al., 1998; Pulido et al., 2000; Zheng and Wang, 2001; Özgen et al., 2006). Phenolic compounds have been recognized as powerful antioxidants *in vitro* and have proven to be more potent antioxidants than Vitamin C, E, and carotenoids (Pulido et al., 2000). Authors suggest phenolic antioxidant properties to be mediated by three main mechanisms: 1. scavenging radical species such as ROS/RNS; 2. suppressing ROS/RNS formation by the inhibition of several enzymes or chelating trace metals involved in the production of free radical production; 3. upregulating or protecting antioxidant defence (Dai and Mumper, 2010).

The structure of phenolic compounds is a key determinant of their radical scavenging and metal chelating activity, and this is referred to as SAR (Aberoumand and Deokule, 2008). Hydroxycinnamic acids have higher antioxidant activity than the corresponding hydroxybenzoic acids, which may be a result of the CH=CH-COOH group, which guarantees the greater capacity to donate hydrogen ions (H+) and stabilize radicals than the carboxyl group. CA (Pal et al., 2011) acts particularly well as a donor of hydrogen atoms, mainly thanks to the extra stability given to the phenoxy radical, resulting from interaction with the adjacent hydroxyl group(s) by hydrogen bonds (Balasundram et al., 2006). By having a hydroxyl in *para*-position relative to the lateral chain, it also easily captures a radical. Besides, it can fluctuate between hydrophilic and lipophilic, which makes it easier for the compound to access areas where there is oxidized vitamin E and, subsequently, can regenerate it (Scott, 1997). *In vitro* and *in vivo* experiments have demonstrated the exceptional antioxidant activity of RA (Rice et al., 2011) against peroxidative damage to biological membranes. RA (Rice et al., 2011) is an ester of CA (Pal et al., 2011) with 3,4-dihydroxyphenyl lactic acid (Petersen, 2013), the major component of polar extracts from many plants of the *Lamiaceae* family (Falé et al., 2009) and, presumably, one of the main compounds responsible for the potent antioxidant activity of *Lamiaceae* plants (Ozgen et al., 2008). The four phenolic hydrogens account for this compound’s ability to modulate free radical scavenging. In combination with two catechol moieties, that provide the suitable polarity for (Rice et al., 2011) to penetrate the lipid bilayers, RA (Rice et al., 2011) has shown to protect against oxidation, without disturbing the molecular structure (Amoah et al., 2016). RA (Rice et al., 2011) protects neurons from OS by significantly reducing H_2_O_2_-induced ROS production and apoptosis cell death, showing the potential application in neurodegenerative diseases, such as PD and Huntingdon’s Disease (HD) (Bhatt et al., 2013). In 2003, Grayer and colleagues demonstrated that the CA (Pal et al., 2011) derivative, nepetoidin B (Nyila et al., 2009), isolated from the aqueous extracts of *P. ecklonii* leaves, has a potent free radical trapping activity (Grayer et al., 2003). Compound (Nyila et al., 2009), nepetoidin B, has been tested, together with three known antioxidants (gallic acid, RA (Rice et al., 2011), and CA (Pal et al., 2011), using the DPPH test. Nepetoidin B (Nyila et al., 2009) showed a higher capacity to capture free radicals than acids (Rice et al., 2011) and (Pal et al., 2011). Nepetoidin A (Van Jaarsveld, 2006) has not been tested enough to gather sufficient evidence. However, even low concentrations of the substance have resulted in a considerable colour loss of a DPPH solution, indicating that nepetoidin A (Van Jaarsveld, 2006) is likely to have strong antioxidant activity as well.

Flavonoid antioxidant activity is attributed to their capability to recapture free radicals and chelate metals (Bilto et al., 2012), as well as their effects on cell signalling and gene expression (Soobrattee et al., 2005). The *in vitro* antioxidant capacity of flavonoids has been intensively studied over the past years and, based on SAR studies, it is predicted that their antioxidant activity depends on its chemical structure, corresponding to the number and position of hydroxyl groups (Amic et al., 2007). *In vivo* antioxidant efficacy of flavonoids appears less in the literature (Pietta, 2000). The antioxidant activity improves notably when C-3' and C-4' positions in ring “B” are occupied by hydroxyl groups (Figure 4). As for ring “A”, phenolic hydroxyl groups contribute somewhat to the antioxidant activity, due to the electrophilic effect of ring “C” (Lin et al., 2014). The presence of ortho-di-hydroxyl (catechol) group on the “B” ring and the double bond between C2-C3 in conjugation with an oxo group at C4 are key structural features of antioxidant flavonoids, since the catechol group stabilizes radical species. Luteolin (Grayer et al., 2003) and its glycosides (e.g. luteolin 7-O-glucoside (Kubínová et al., 2013)) satisfy these structural necessities, therefore, it is not surprising that many luteolin-containing plants possess antioxidant properties, through their ability to scavenge ROS and RNS (López-Lázaro, 2009). In a study of the copper chelating properties of luteolin-7-*O*-glucoside (Kubínová et al., 2013) and luteolin (Grayer et al., 2003)**,** the ortho-3’,4’-dihydroxy substitution in the B-ring, in the case of luteolin (Grayer et al., 2003)**,** was suggested as being important for copper chelation, thereby influencing its antioxidant activity (Brown and Rice-Evans, 1998).

There is also evidence in the literature that simultaneous hydroxylation of C3 and C5 flavonoids is another important structural feature involved in maximizing the potential for free radical scavenging, in determining antioxidant activity (Bors et al., 1990; Soobrattee et al., 2005). Besides, the existence of a portion of sugar in the C8 position of vitexin (Gaspar-Marques et al., 2006) significantly decreases the antioxidant efficacy of this compound compared to its non-glycosylated derivative (Simões et al., 2010) (Soobrattee et al., 2005). The antioxidant activity of the flavones salvigenin (Van Zyl et al., 2008), cirsimaritin (Nyila et al., 2012)**,** and genkwanin (Zschocke et al., 2000) have been evaluated by the qualitative tests of DPPH and the discoloration of ß-carotene. They all tested negative for DPPH, which means that they do not pick-up radicals by this method. Salvigenin (Van Zyl et al., 2008) was the only one to test positive for beta-carotene bleaching, which may indicate preventive antioxidant activity, possibly related to the absorption of UV radiation (Gaspar-Marques et al., 2006). Preventive antioxidants can be compounds with the ability to absorb UV rays, superoxide dismutase enzymes, catalases, and peroxidases, or compounds with the ability to chelate or reduce transition metals (Scott, 1997). The use of flavones in the treatment of Alzheimer’s disease focuses on the inflammation process underlying the progression of the disease. This therapeutic approach is based on the preventive action of flavones in the face of OS and consequent inflammation by acting as antioxidants by capturing free radicals.

### Anti-inflammatory

Several studies have shown that triterpenoids significantly suppress chronic inflammation by modulating proinflammatory mediators. The anti-inflammatory effects of pentacyclic triterpenoids are largely ascribed to their ability to inhibit molecular targets such as 5-lipoxygenase (LOX), inducible nitric oxide synthase (iNOS), cyclooxygenase (COX) - 2, and nuclear factor-kappa B (NF-κB) activities (Yap and Lim, 2015). The anti-inflammatory effects of UA (Lukhoba et al., 2006) have been attributed to its ability to suppress nuclear factor-kappa B (NF-κB) activation, which, together with NF-AT (nuclear factor of activated T cells) and AP-1 (activator protein-1), regulate inflammatory genes (Checker et al., 2012). Another potential application of this compound could be in the treatment of osteoarthritis since the activation of NF-kB is critical in the pathophysiology of osteoarthritis (Gabay et al., 2010). The potential anti-inflammatory activity of sugiol (de Albuquerque et al., 2007) and the relationship between signal transduction and inflammatory cytokines was evaluated *in vitro* (Chao et al., 2005). A dose of 30 µM of sugiol effectively inhibited the production of pro-inflammatory cytokines, prointerleukin-1beta, IL-1β, and tumour necrosis factor-alpha (TNF-α), suggesting that sugiol (de Albuquerque et al., 2007) is bioactive against inflammation. The authors suggested that the efficacy of sugiol (de Albuquerque et al., 2007) in inhibiting inflammatory cytokines IL-1ß and TNF-α could be attributed to a reduction of ROS, which in turn causes a decrease in the phosphorylation of mitogen-activated protein kinases (MAPKs).

The anti-inflammatory properties of RA (Rice et al., 2011) are thought to be based on the inhibition of LOX and COX, on the interference with the complement cascade and the inhibition of expression of inflammatory cytokines. Another study has shown that CA (Pal et al., 2011) derivatives exert anti-inflammatory action *in vitro* and *in vivo* and their action is mediated, at least partially, by NO recapture (Da Cunha et al., 2004). Nakanishi and colleagues reported potent inhibition of xanthine oxidase by both Nepetoidin A (Van Jaarsveld, 2006) and B (Nyila et al., 2009) and particularly by nepetedoin B (Nyila et al., 2009), suggesting that this compound could have the potential for the control of hyperuricemia in human gout (Nakanishi et al., 1990).

The anti-inflammatory effect of phenolic compounds is related to the ability to modulate the expression of pro-inflammatory enzymes such as phospholipase A2, nitric oxide synthase (NOS), COX, and LOX. Inhibition of these enzymes by flavonoids reduces the production of arachidonic acid, prostaglandins (PG), LTs, and NO, crucial mediators of inflammation. In general, flavones have a greater inhibitory effect on NO production than flavonols (Kim et al., 2004). Different flavonoids, such as quercetin, apigenin (Simões et al., 2010) and luteolin (Grayer et al., 2003), have been reported to possess anti-inflammatory and analgesic effects (Kumar and Pandey, 2013). Apigenin (Simões et al., 2010) showed strong anti-inflammatory activity through inhibition of NO and iNOS production, and inhibition of COX-2 expression. Inhibition of iNOS and NO production is also attributed to luteolin (Grayer et al., 2003) (Choi et al., 2014). Apigenin (Simões et al., 2010) and luteolin (Grayer et al., 2003) also inhibit interleukin (IL)-5, which promotes the growth and survival of eosinophils and plays an important role in allergic inflammation associated with eosinophilia (Packer et al., 2004). In an *in vitro* study, both flavones showed potent inhibition of IL-4 and IL-13 synthesis (Hirano et al., 2004), and both have an inhibitory action on LOX and pro-inflammatory cytokines TNF-α and IL-1 (Lago et al., 2014). On a structural level, the requirements for the anti-inflammatory activity of the flavonoids include unsaturation in the C-ring (between C2 and C3); the number and position of hydroxyl groups (e.g., the catechol group in the B-ring); the carbonyl group in C4; and the non-glycosylation of the molecule. However, compounds that do not have these structural characteristics also exhibit anti-inflammatory activity, affecting enzymes of the inflammatory cascade (Lago et al., 2014). Data in the literature strongly suggest that the double bond between C2 and C3 is crucial for inhibiting NO production and that hydroxyl substitutions in the A and B rings influence inhibitory activity **(** Figure 4 **)**. Hydroxylation on positions 5- and/or 7- of the A-ring, and in positions 3'- and/or 4'- of the B-ring provide favourable results for inhibition of production, the opposite if hydroxylation is in carbon 3 (C-ring) (Kim et al., 1999). Apigenin (Simões et al., 2010) and luteolin (Grayer et al., 2003) are among the flavonoids cited as the most active inhibitors. The anti-inflammatory effect of luteolin (Grayer et al., 2003), its glucosides and plants containing luteolin (Grayer et al., 2003) have been tested *in vitro* and *in vivo* (López-Lázaro, 2009). In an *in vitro* SAR study, luteolin (Grayer et al., 2003) showed high inhibitory activity of thromboxane and LT synthesis, and in particular against the enzyme activity of LTs. Cinaroside (Kubínová et al., 2013) (luteolin-7-*O*-*ß*glucoside) showed only moderate inhibitory activity against both enzyme synthesis pathways (Odontuya et al., 2005). These results support the idea that the hydroxyl substitute in the C5 position and the non-glycosylation of the molecule contribute significantly to the anti-inflammatory activity of the flavonoids. *In vivo* studies have shown that luteolin (Grayer et al., 2003) effectively protects mouse induced LPS lethality, suggesting the application of this compound as a potential therapeutic agent in septic decay treatment (Kuo et al., 2011; Chen et al., 2014).

### Enzyme Inhibition

Tyrosinase is one of the keys enzymes in the biosynthesis of melanin, the pigment responsible for determining skin and hair colour. The inhibition of tyrosinase is one of the major strategies to treat skin hyperpigmentation, one of the common skin complaints that affect people of all skin types. Inhibitors of the tyrosinase enzyme, such as hydroquinone, kojic acid, and azelaic acid, have been used to treat hyperpigmentation disorders but despite their efficacy, many of these agents are frequently reported to have numerous limitations, such as high cytotoxicity, poor skin penetration, and low stability in formulations (Otang-Mbeng and Sagbo, 2020). Therefore, there has been a growing demand for products that act safely and effectively when inhibiting enzymatic oxidation, to prevent hyperpigmentation (Nerya et al., 2004). One of the traditional preparations, a paste made from the leaves of *P. ecklonii*, is used in Zimbabwe for skin diseases and skin hyperpigmentation problems.

*P. ecklonii* ethyl acetate extract and its isolated compounds, parviflorone D (Salim et al., 2008) and F (Srancikova et al., 2013), were tested for their tyrosinase inhibitory action in comparison to kojic acid. The concentration at which half the tyrosinase activity was inhibited (IC_50_) by the extract was 61.7 ± 2.7 µg/ml. During cytotoxicity evaluation, compounds (Salim et al., 2008) and (Srancikova et al., 2013), were toxic against monkey kidney Vero cell lines, as shown by their IC_50_ values (Nyila et al., 2009). Nevertheless, the activity demonstrated by the raw extract of *P. ecklonii* in the tyrosinase test, together with its antibacterial activity against *S. aureus*, helps to justify the traditional use of the plant in skin-related diseases (Lukhoba et al., 2006). In a more recent study, the high chelating ability of abietane diterpenes, found in *P. ecklonii,* was attributed to the observed anti-tyrosinase activity, *in vitro*, and was considered almost as efficient as kojic acid, the positive control. The combination of polyphenolic compounds, such as quercetin, with abietane diterpenes, has shown a synergistic effect that promotes both anti-tyrosinase and antioxidant activity, suitable for skin treatment, such as, anti-pigmentation (Andrade et al., 2021).

Acetylcholinesterase (AChE) is the enzyme that catalyses the hydrolysis of the neurotransmitter acetylcholine (ACh) (Dvir et al., 2010). Nowadays, the most effective therapy for Alzheimer’s disease (AD) consists of increasing the levels of ACh through the inhibition of AChE activity (Falé et al., 2009). The literature indicates that terpenoids and, in particular, some diterpenoids, may have anti-acetylcholinesterase activity (Dvir et al., 2010). To date, no references to the inhibitory effect of AChE by diterpenes with an abietane skeleton isolated from *P. ecklonii* have been found in the literature.

AChE is the target of cholinesterase (ChE) inhibitors used when addressing the cholinergic deficit in AD patients. The leading AD therapeutics involve AChE inhibitors, which produce an increase of the acetylcholine concentrations in the synaptic cleft, enhancing the cholinergic transmission. Despite decades of research, current pharmacotherapeutic options for AD are still very limited and represent an area of need that is currently unmet. Studies indicate that species of the *Lamiaceae* family are a bountiful source of varying natural AChE inhibitors and antioxidants that could be useful in the prevention and treatment of AD and other related diseases (Vladimir-Knežević et al., 2014). In a study with *P. barbatus*, the presence of RA (Rice et al., 2011), a compound which is also found in *P. ecklonii*, has been attributed to the antioxidant activity found *in vitro* and the inhibition of AChE, where high inhibition activity was demonstrated in the decoction (31% inhibition). The authors also analysed other *Plectranthus* spp., *P. ecklonii*, *P. fructicosus*, *P. lanuginosus*, and *P. verticillatus*, where they compared the RA (Rice et al., 2011) content of the plants. *P. ecklonii* was the species studied that gave the highest inhibition activity (62.8%) (Falé et al., 2009). In a study looking for new treatment strategies for AD, the *in vitro* AChE inhibition, antioxidant activity, and bioactive components of five different spp. of *Plectranthus* (*P. ecklonii* including) were investigated. The main components of the aqueous extracts, rosmarinic (Rice et al., 2011), chlorogenic (Chassagne and Morgan, 2020) and caffeic (Pal et al., 2011) acids, were quantified. The decoctions showed high AChE inhibitory and antioxidant activities for *P. ecklonii* and *P. saccatus* (Gomes et al., 2012). According to these studies, the aqueous extracts and decoction method is the best way to evaluate the AChE activity of *P. ecklonii*. It has also been stated that the most active extracts were obtained from the leaves as opposed to the flowers (Falé et al., 2009). The activity of other enzymes, such as collagenase inhibition, in aqueous extracts of *P. ecklonii*, has recently been established with rosmarinic (Rice et al., 2011) being attributed to promoting the highest amount of biological activity at 4.5%. However, the presence of CA (Pal et al., 2011) in the extracts could also be considered accountable for the increased activity and further studies would be required to positively identify the compounds responsible for the enzyme inhibition (Andrade et al., 2021). RA’s (Rice et al., 2011) inhibitory activity of the enzyme glycosyltransferase (GTF) has been highlighted (Figueiredo et al., 2010) and the presence of this compound has also been linked to the observed effects of AChE inhibition and antioxidant activity (Falé et al., 2009). The flavones vitexin (Gaspar-Marques et al., 2006), isovitexin (Amoah et al., 2016) and naturally occurring C-glycosylated derivatives of apigenin (Simões et al., 2010) have demonstrated anti-AD activity (Choi et al., 2014), again promptly the need for further studies of this species to corroborate these findings.

## Conclusion

With the growing acceptance of alternative forms of health care, such as traditional medicine, new requirements are also emerging. Compound screening is needed to clarify which molecules are responsible for biological activities, to scientifically validate popular plant uses. Secondary metabolites found in plants provide an immeasurable wealth of structurarly diverse compounds with associated bioactivities The genus *Plectranthus*, for its diverse ethnobotanical applications and the several biological effects (antimicrobial, antioxidant, anti-inflammatory and anti-tumour), has been suggested as a promising source for the discovery of bioactive compounds. In this sense, the isolation of secondary metabolites from *Plectranthus* species and the understanding of the origin of their therapeutic properties is imperative and can guarantee effective and safe use. In this work not only have some of the traditional uses of *P. ecklonii* been validated, but the potential of this plant as a prospective source of new drug leads has been demonstrated. From the species *P. ecklonii* Benth., 28 compounds have been isolated to date. As observed in other *Plectranthus* species studied over the years, the predominant classes are terpenes and phenolic compounds. Eight diterpenes were identified in *P. ecklonii*, four of them being diterpenes with an abietane skeleton (**1**, **2**, **9**, and **27**), two being triterpenes (**12** and **13**) and two being identified as sterols (**14** and **15**). Regarding phenolic compounds, twelve flavones (**19-24**, **26** and **28**-**31**) and one flavanone (Costa et al., 2018) were isolated, in addition to CA (Pal et al., 2011) and four of its derivatives (**3**, **4**, **5**, and **16**). The literature reports RA (Rice et al., 2011) as the predominant compound in the aqueous extracts of *P. ecklonii*. Phytochemical studies have reported the isolation of two isomeric *o*-quinones, ecklonoquinones A (Śliwiński et al., 2020) and B (Andrade et al., 2018) from *P. ecklonii* which have not been analysed in this work because no reference was found in the literature concerning any bioactivities (Uchida et al., 1980). Among the diterpenes, the most recent studies emphasise the major abietane diterpenoid, parviflorone D (Salim et al., 2008), for its potential in cancer therapy, particularly when combined with nanotechnology, and sugiol (de Albuquerque et al., 2007) has also demonstrated its anticancer potential as an effective topoisomerase 1 inhibitor. The main phenolic compound, rosmarinic acid (Rice et al., 2011), mainly exhibited antioxidant and anti-inflammatory activity, although photoprotective and melanogenic properties have also been described. Anti-inflammatory activity has also been attributed to sugiol (de Albuquerque et al., 2007), caffeic acid (Pal et al., 2011), apigenin (Simões et al., 2010) and luteolin (Grayer et al., 2003). The diterpenes parviflorone D (Salim et al., 2008), E (Abdel-Mogib et al., 2002), F (Srancikova et al., 2013) and sugiol (de Albuquerque et al., 2007) all showed antioxidant activity as well as oleanolic acid (Dellar et al., 1996)**,** caffeic acid (Pal et al., 2011) and Nepetoidin B (Nyila et al., 2009).The antimicrobial activity of *Plectranthus ecklonii* has been attributed to parviflorone D (Salim et al., 2008) and F (Srancikova et al., 2013), the latter also showing similar antimalarial activity similar to that of chloroquine. Adittionally, (2) and (Abdel-Mogib et al., 2002) were reported to be more effective than quinine. As an ornamental plant, with multiple compounds showing anticancer activity, *P. ecklonii* could become an accessible source for antitumour drugs. Since *P. ecklonii* is a common species of South Africa, in terms of economising and the sustainable use of resources from natural products, *P. ecklonii* could be a legitamate alternative to current antitumour drugs. However, the lack of data and information on possible side effects and saftey of this species warrants further investigation, to assess the safety of this plant for clinical and therapeutic use.
